# Experimental techniques and terminology in gas‐phase ion spectroscopy

**DOI:** 10.1002/jms.4826

**Published:** 2022-04-17

**Authors:** Aleksandr Pereverzev, Jana Roithová

**Affiliations:** ^1^ Institute for Molecules and Materials Radboud University Nijmegen The Netherlands

**Keywords:** action spectroscopy, ion spectroscopy, IRMPD, LIICG, LIR, luminescence, mass spectrometry, photodissociation, tagging

## Abstract

This perspective gives an overview of the action spectroscopy methods for measurements of electronic, vibrational, and rotational spectra of mass‐selected ions in the gas phase. We classify and give a short overview of the existing experimental approaches in this field. There is currently a plethora of names used for, essentially, the same techniques. Hence within this overview, we scrutinized the notations and suggested terms to be generally used. The selection was either driven by making the name unique and straightforward or the term being the most broadly used one. We believe that a simplification and a unification of the notation in ion spectroscopy can make this field better accessible for experts outside the mass spectrometry community where the applications of gas‐phase action ion spectroscopy can make a large impact.

## 1 INTRODUCTION

Spectroscopy of isolated ions in the gas phase provides information on intrinsic properties of these species free from any possible effects of the surrounding media. First reports on spectroscopy of mass‐selected ions date back to the early 1970s.^1^ It started as wavelength‐dependent photodissociation experiments in the visible (Vis) spectral range. Soon afterward, methods to measure vibrational spectra started to be developed as well.^2^ In the beginning, photodissociation spectroscopy of mass‐selected ions was reserved only to a few laboratories because the experimental setups required custom mass spectrometers and lasers or optical parametric oscillators (OPOs). Nowadays, technical developments in all directions make action ion spectroscopy setups available to a steeply increasing number of laboratories. In addition, photodissociation spectroscopy of ions in the infrared (IR) range is available almost at demand at free‐electron laser (FEL) facilities. As a result, photodissociation spectroscopy became an integral part of mass spectrometry investigations of ions. It substantially increased our knowledge about organic, inorganic, metal organic, or biological molecules in the gas phase.^3^, ^4^

During the last decades, researchers developed and improved numerous experimental methods for measuring gas‐phase spectra of ions. This was often associated with a modification of terminology in this field. As a result, the nomenclature became smeared and sometimes unclear. A quick overview reveals that often many naming variations exist describing essentially the same approach. In this paper, we have outlined 27 different spectroscopic methods used in the gas‐phase action ion spectroscopy, but the number of naming variations for these techniques that we found in literature is over 200 (see Supporting Information). The differences in naming of methods in the ion spectroscopy are often intuitive and aimed to highlight certain aspects of a given spectroscopic approach. This can be useful and completely understood within the community of researchers developing these methods. However, with increasing maturity of this type of spectroscopy, the aim should be to make gas‐phase action ion spectroscopy widely known and accepted as a standard tool for investigation of ions. For broad audience, this requires a clear vocabulary.

In this paper, we aimed to collect and classify spectroscopic methods used to characterize mass‐selected ions in the gas phase based on the action consequences of light absorption. We have collected and sorted the terms that are used in the field of ion spectroscopy according to the methods they refer to and propose a nomenclature that clearly differentiates between methods. We propose that the names of experimental techniques specify the nature of the spectroscopically probed transitions (electronic, vibrational, or rotational), the spectroscopic excitation scheme, and the detection method (photodissociation, light‐induced reactions [LIR], luminescence, etc.). The selected terms suggested to be generally used are highlighted in the **bold** font. We list only a few alternative terms in tables, and a complete list of currently used terms with citations can be found in the corresponding supplementary tables in the Supporting Information. This article does not aim to cover contributions of researchers in this field, and it does not aim to explain details of all methods. For the latter, we refer the readers to numerous reviews that are devoted to the methods themselves.^4^, ^5^, ^6^, ^7^, ^8^, ^9^, ^10^, ^11^, ^12^, ^13^, ^14^ We want to also stress that most of the spectroscopic methods in gas‐phase action spectroscopy originate from the previous research of neutral molecules in the gas phase. Here, however, we focus only on the gas‐phase action spectroscopy of mass‐selected ions and leave out entirely the classification of gas‐phase action spectroscopy of neutral molecules.

In the first part, we discuss general terms in the gas‐phase action spectroscopy methods. In the second and third parts, we present the classification of methods of the gas‐phase electronic and vibrational photodissociation spectroscopy of bare ions and of complexes of the ions with an atomic or a molecular tag, respectively. The fourth part describes alternative detection principles for recording action ion spectra in the gas phase. The fifth part is dedicated to rotational action ion spectroscopy methods. The sixth part briefly mentions possible extensions of the existing experiments that, however, do not alter spectroscopic schemes. Finally, in the seventh part, we discuss the names of spectra recorded by all of the methods described above.

## 2 GENERAL TERMS FOR ACTION SPECTROSCOPY OF MASS‐SELECTED IONS

### 2.1 Gas‐phase action spectroscopy

We will focus solely on spectroscopy of ions in the gas phase (within a mass spectrometer), usually referred to as ion spectroscopy or gas‐phase ion spectroscopy. The more general term, gas‐phase spectroscopy, refers to ions as well as neutral species in the gas phase (the latter is not reviewed here).

Gas‐phase action spectroscopy of ions, non‐covalently bound ion–ion or ion–neutral complexes, solvated ion clusters, etc. usually does not rely on a direct detection of light absorption. Given an extremely low concentration of ions in a mass spectrometer as compared with typical concentrations in the condensed phase, the measurement of a direct absorption of light is nearly impossible. Hence, other effects of photon absorption are usually detected, photodissociation being the most widespread but not the only one. Other methods include luminescence, reactivity promotion, etc. (see following text for details). Because of detecting of an action consequence of the photon absorption, the techniques are often referred to as action spectroscopy or consequence spectroscopy (Tables 1 and S1). We suggest using **gas‐phase action ion spectroscopy** as a general term that encompasses all these methods. The terms “gas‐phase” and “ion” stress that it is spectroscopy of mass‐selected ions, which however is often clear from the context, and then **action spectroscopy** is an appropriate term. Similarly for all the following terms, adding “gas‐phase” and “ion” to the names of the techniques might be redundant.

**TABLE 1:** General terms for action ion spectroscopy in the gas‐phase (see Table S1 for the complete list with the references)

Name of the technique	Other terms used in literature
Gas‐phase **action** ion **spectroscopy**	Action spectroscopy Ion spectroscopy Gas‐phase spectroscopy Gas‐phase action spectroscopy Gas‐phase ion spectroscopy
**Photodissociation** ion **spectroscopy**	Photodissociation spectroscopy Photofragment spectroscopy Photofragmentation spectroscopy Photodissociation action spectroscopy Laser dissociation spectroscopy
**Cryogenic** gas‐phase **action** ion **spectroscopy** **Cryogenic photodissociation spectroscopy**	Cold ion spectroscopy (CIS) Gas‐phase cold ion spectroscopy (CIS) Cryogenic ion spectroscopy Cryo‐cooled ion spectroscopy Cryo‐cooled gas‐phase spectroscopy
**Electronic** gas‐phase **action** ion **spectroscopy**	Electronic spectroscopy UV spectroscopy UV photodissociation (UVPD) spectroscopy Gas‐phase electronic spectroscopy UV cold ion spectroscopy (CIS)
**Vibrational** gas‐phase **action** ion **spectroscopy**	Vibrational spectroscopy IR spectroscopy Gas‐phase vibrational spectroscopy IR photodissociation (IRPD) spectroscopy Cryogenic IR spectroscopy
**Rotational** gas‐phase **action** ion **spectroscopy**	Rotational spectroscopy Rotational action spectroscopy Microwave spectroscopy
Multi‐resonance spectroscopic techniques	Double‐resonance spectroscopy IR–UV photodissociation spectroscopy IR–UV double‐resonance spectroscopy IR–UV double‐resonance photofragment spectroscopy IR–UV double‐resonance cold ion spectroscopy (CIS)
Isomer/conformer selectivity	Conformation‐specific spectroscopy Conformer‐selective cold ion spectroscopy (CIS) Conformation‐selective electronic spectroscopy Conformer‐selective vibrational spectroscopy Conformer‐selective cryogenic IR spectroscopy

### 2.2 Specification of the detection method

Although the term **gas‐phase action ion spectroscopy** encompasses all possible detection techniques, each detection method may affect the resulting spectrum in its own unique way; hence, a more specific notation must be used, unambiguously highlighting the detection principle.

Photodissociation of isolated ions following the light absorption (provided that the energy of the absorbed photons is larger than the bond dissociation threshold) is, probably, one of the most straightforward and experimentally least demanding detection techniques discussed herein. Therefore, most gas‐phase action ion spectroscopy methods rely on the detection of charged dissociation products or on the depletion of the parent ion signal following absorption of one or multiple photons. This also includes photoinduced electron detachment from anions. The principle of the photon absorption detection by photoinduced dissociation is often emphasized by “fragmentation,” “dissociation,” “photofragment,” “photofragmentation,” “photo‐induced dissociation,” or similar adjectives to the name of the method (Tables 1 and S1). These terms originate from mass spectrometry terminology and can be used more or less interchangeably. For the sake of simplicity and uniformity, we propose to use the term **photodissociation** and refer to the corresponding techniques as **photodissociation ion spectroscopy**. Other possible detection principles are discussed in Section 4. For these methods, the corresponding term must be used instead of “photodissociation” to unambiguously indicate the experimental methods.

### 2.3 Cryogenic gas‐phase action ion spectroscopy

Many gas‐phase photodissociation spectroscopic techniques are based on the cooling of the ions to low temperatures. The cooling simplifies the observed spectra because it reduces conformational heterogeneity and spectral congestion in the UV/Vis range by confining ions in their ground vibrational state. This feature is sometimes emphasized by adding terms such as “cryogenic,” “cryocooled,” “cold ion,” etc. (Tables 1 and S1) to the name of the technique. We propose to use the general term **cryogenic (gas‐phase) action ion spectroscopy** or more specifically **cryogenic photodissociation ion spectroscopy**.

### 2.4 Specification of the spectral range

The name of the spectroscopic technique should, ideally, specify which type of transitions are spectroscopically probed. Therefore, one should correctly speak of **electronic**, **vibrational**, or **rotational gas‐phase action (photodissociation) ion spectroscopy**. However, even classical condensed‐phase spectroscopies are commonly referred to by the working spectral range (such as IR or UV spectroscopy). We thus propose to use analogous notation, just with an addition of the term specifying the detection method (see Tables 1, 2, 3, 4, 5 and S1–S6).

### 2.5 Specifying the isomer/conformer selectivity

Many polyatomic ions are present in the gas‐phase as a mixture of several isomers/conformers. The possibility to differentiate between multiple isomer/conformer forms is often achieved by using additional laser(s) to assign individual spectral features to each geometric configuration. The latter is usually highlighted by adding term “conformer‐selective” (or similar) to the general name of the method, for example, “conformer‐selective gas‐phase vibrational photodissociation ion spectroscopy.” In the proposed naming of the methods, each of them has a name that unambiguously identifies the method, thus removing the necessity to use additional clarification. However, when one needs to specify this feature, we suggest using the term **isomer/conformer‐selective** for the techniques and the term **isomer/conformer‐specific** for the corresponding spectra.

## 3 CLASSIFICATION OF THE GAS‐PHASE PHOTODISSOCIATION ION SPECTROSCOPY METHODS FOR BARE IONS

### 3.1 Electronic photodissociation spectroscopy of bare ions

**SCHEME 1:**
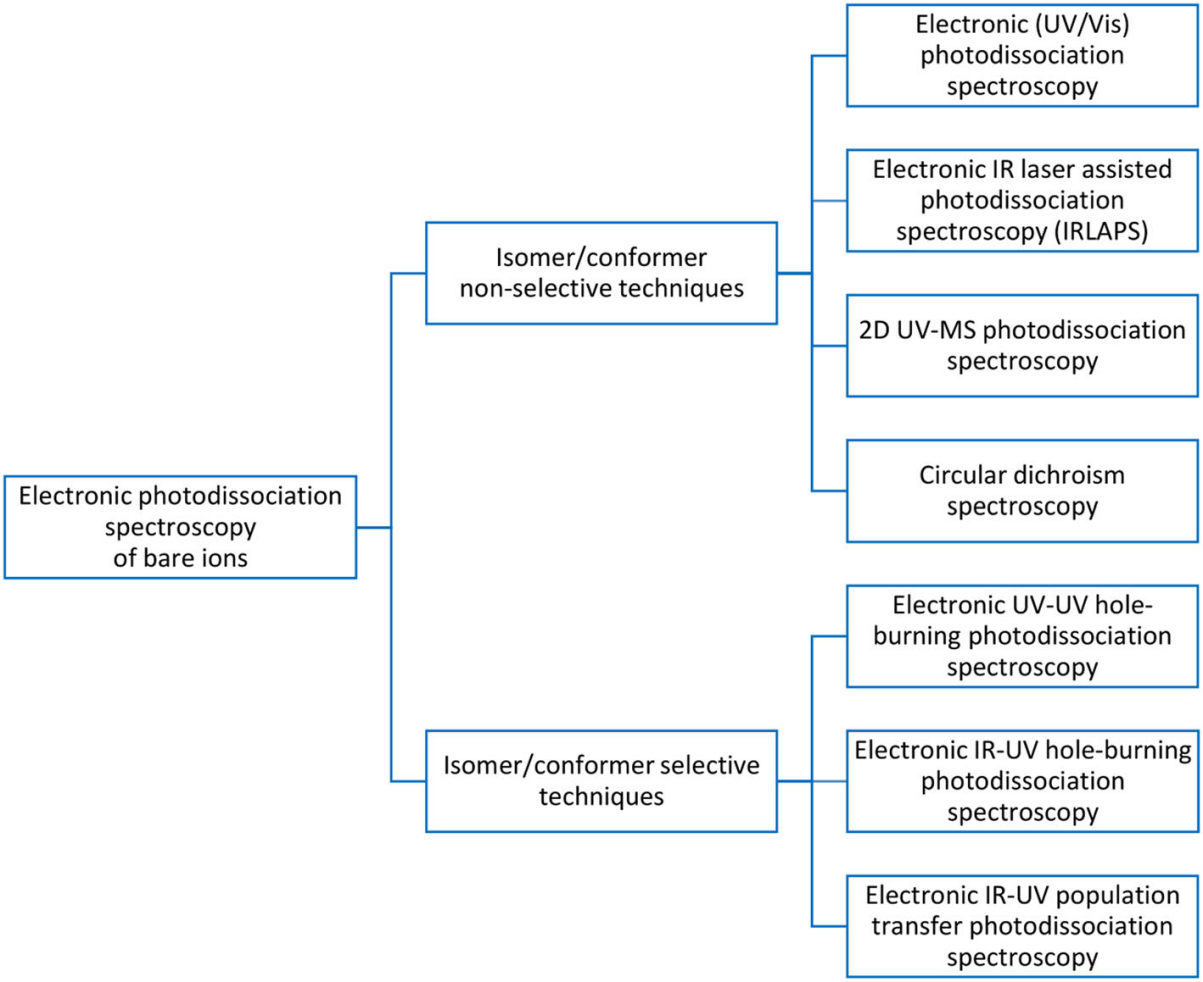
Classification of the methods for electronic photodissociation spectroscopy of bare ions

Scheme 1 summarizes the spectroscopic techniques for obtaining gas‐phase electronic photodissociation spectra of bare ions. In the following, we provide brief description of these methods. The terms used in the field are summarized in Table 2 (for the full list of terms see Table S2).

**TABLE 2:** Terms for electronic photodissociation spectroscopy of bare ions (see Table S2 for the complete list and the references)

Name of the technique	Other terms used in literature
**Isomer/conformer non‐selective techniques**	
**Electronic (UV/Vis) photodissociation** ion **spectroscopy**	Electronic spectroscopy UV spectroscopy UV/Vis action spectroscopy Gas‐phase UV spectroscopy UV photodissociation (UVPD) spectroscopy
**Electronic infrared laser‐assisted photodissociation** ion **spectroscopy (IRLAPS)**	Infrared laser‐assisted photofragment spectroscopy Two‐laser multiphoton dissociation spectroscopy
**2D UV‐MS photodissociation** ion **spectroscopy**	2D UV‐MS spectroscopy 2D UV‐MS cold ion spectroscopy (CIS) UV fragmentation spectroscopy–mass spectrometry (UV–MS)
**Circular dichroism ion spectroscopy**	Electronic circular dichroism (CD) ion spectroscopy Circular dichroism (CD) spectroscopy
**Isomer/conformer selective techniques**	
**Electronic UV–UV hole‐burning photodissociation** ion **spectroscopy**	UV–UV hole‐burning spectroscopy UV–UV double‐resonance spectroscopy
**Electronic IR–UV hole‐burning photodissociation** ion **spectroscopy**	IR–UV hole‐burning spectroscopy
**Electronic IR–UV population transfer photodissociation** ion **spectroscopy**	IR–UV hole‐filling spectroscopy IR–UV population transfer hole‐filling spectroscopy

### 3.2 Electronic (UV/Vis) photodissociation ion spectroscopy

Gas‐phase electronic photodissociation ion spectroscopy is a rather widespread technique, because electronic excitation of ions often leads to their dissociation either from the excited or from the ground electronic state following internal energy conversion. The spectra can be recorded using tunable tabletop lasers while monitoring the intensity of either one of the photodissociation products (or their integral intensity) or the intensity of the parent ion as a function of the laser wavelength.^1^ The technique is usually referred to as UV/Vis photodissociation spectroscopy. The spectra recorded at room temperature are usually unstructured due to the thermal broadening. In order to achieve a good resolution, the ions should be in their ground vibrational state. Therefore, the spectra are typically recorded at cryogenic temperatures, which requires dedicated instruments.

### 3.3 Electronic infrared laser‐assisted photodissociation ion spectroscopy (IRLAPS)

Photodissociation of large biomolecular ions is often in competition with other energy relaxation processes, which reduce the photodissociation yields. This limitation can be overcome by additional excitation of the ions by multiple IR photons.^15^ The power of the IR laser (typically CO_2_) is chosen such that no dissociation occurs in the absence of the UV/Vis excitation. Additional energy from the absorbed IR photons significantly increases the dissociation yield. In the first reports of this method, the IR excitation by a continuous laser preceded or was simultaneous with the electronic one. However, later in the investigations of the electronic spectra of cold ions, the electronic excitation always preceded the IR excitation with a pulsed laser. The electronic excitation must be resonant with transitions from the ground vibrational state.^16^ This method of recording electronic spectra of ions is referred to as infrared laser‐assisted photofragment spectroscopy (IRLAPS); however, this term is also used sometimes for two‐color IR multiple photon dissociation spectroscopy. In order to avoid any confusion, we suggest using the term **electronic infrared laser‐assisted photodissociation ion spectroscopy**.

### 3.4 2D UV‐MS photodissociation ion spectroscopy

The coupling of electronic photodissociation spectroscopy with high‐resolution mass spectrometry is sometimes referred to as 2D UV‐MS spectroscopy.^17^ A use of orbitrap, ICR, or TOF mass spectrometers allows simultaneous detection of all dissociation products as a function of a laser wavelength. The relative abundance of different dissociation products depends on the dissociation pathways available at the energy of the excited electronic state. Hence, the fragmentation pattern depends on the energy of the absorbed photon. Therefore, the 2D map of the dissociation patterns as the function of the photon energy can provide a detailed information on ion isomers and even on conformers. Differences in the dissociation patterns and electronic absorption spectra allow for an accurate quantification of mixtures of isomeric species.

### 3.5 Circular dichroism ion spectroscopy

Recently, it has been demonstrated that electronic circular dichroism (CD) spectra could be recorded by electron detachment from mass‐selected anions in an ion trap. This method reflects the differences of the dissociation yields induced by left‐ or right‐circularly polarized light.^18^

### 3.6 Electronic UV–UV hole‐burning photodissociation ion spectroscopy

The use of two lasers opens a possibility to measure individual UV/Vis spectra of different isobaric ions—isomers, or conformers—in a mixture. The first laser wavelength is tuned to a specific electronic transition of one isomer, whereas the second UV laser is scanned to measure the electronic spectrum of the remaining ions. Until recently, this technique was never applied for ions in the ion trap because of the difficulty to distinguish ion signals produced by the pump and the probe lasers. Hence, this technique requires special detection methods, such as the extraction of the photodissociation product ions from the quadrupole ion trap immediately after the laser irradiation^19^ or the detection of the neutral fragments ^20^ (the latter removes all rf‐related limitations in QIT). The difference between the spectra measured with the first laser on and off results in the electronic spectrum of one specific isomer/conformer. These experiments are usually called “hole‐burning” spectroscopy. “Hole‐burning” refers to the depletion of the population of a given isomer/conformer.

### 3.7 Electronic IR–UV hole‐burning photodissociation ion spectroscopy

Alternatively, one can use an IR laser instead of the UV laser and excite a specific transition of a selected isomer/conformer. The first “pump” IR laser wavelength is tuned to a vibrational transition unique for a single isomer/conformer to selectively excite it. Such a selective excitation heats up the single cold isomer internally and significantly broadens the corresponding UV spectrum. As the result, all sharp UV transitions originating from the ground vibrational state of the IR‐excited isomer/conformer disappear from the UV spectrum of the ion ensemble. The spectrum will thus show only the UV transitions of the ions that did not absorb the IR photons.^21^ The depletion of the spectral signature of the given ions is referred to as “hole‐burning.”

### 3.8 Electronic IR–UV population transfer photodissociation ion spectroscopy

IR preexcitation can be also used to study conformational changes of the ions happening after the IR photon absorption. If the energy of the absorbed IR photon is sufficient for overcoming of the energy barriers for isomerization, the initial population of the excited isomers may be redistributed among other various isomers/conformers. After a time delay sufficient for the rearrangements and for the re‐cooling of the IR excited ions, the new conformer/isomer distribution is tested by measuring UV absorption. The difference between the UV spectra with and without the IR excitation reflects the changes in the relative populations of the various isomers/conformers. This type of experiment is referred to as “hole‐filling” or “population transfer.”^22^ Both terms concern the change of the populations of isomers/conformers. We suggest using the term **electronic IR–UV population transfer ion spectroscopy** as it better reflects the essence of the method.

Note that in double‐resonance experiments, the notation with the spectral range and the indication of the type of the experiment (such as electronic UV–UV hole‐burning photodissociation ion spectroscopy) is the preferred notation.

## 4 VIBRATIONAL PHOTODISSOCIATION SPECTROSCOPY OF BARE IONS

In order to obtain a vibrational spectrum of an ion, absorption of an IR photon must lead to a dissociation. However, absorption of a single IR photon is almost never sufficient to induce a dissociation of covalent bonds. There are three general strategies to overcome this limitation: (1) using a high‐power laser and work in a multiple photon absorption regime (IRMPD spectroscopy), (2) combining IR photon absorption with subsequent UV photon absorption that induces the desired dissociation (so‐called “double‐resonance” techniques), and (3) preparing loosely bound complexes with inert molecules with binding energies below the energy of one IR photon (tagging techniques). The latter approach will be discussed in the next section. Some techniques can affect the resulting IR spectrum. Therefore, it is advisable to clearly specify the type of the technique in the name of spectroscopy.

Whereas IRMPD (infrared multiple photon dissociation) spectroscopy is a well‐established notation, the alternative, so‐called double‐ or multi‐resonance techniques for measuring IR spectra are often denoted just by the operational spectral range of the excitations used, such as infrared–ultraviolet (IR–UV) photodissociation ion spectroscopy (see Table S3 for other variants). However, this notation can be confusing, because it can refer also to electronic spectroscopy (the most significant example is IR–UV population transfer spectroscopy). Similarly, the names specifying isomer/conformer selectivity of the method, yet lacking a clear reference to the method itself, should be avoided. The complete list of such names is in Table S3. In the following, we provide description of all the techniques used for vibrational photodissociation spectroscopy of bare ions and suggest the names that unambiguously refer to the corresponding technique while trying to keep consistency.

**SCHEME 2:**
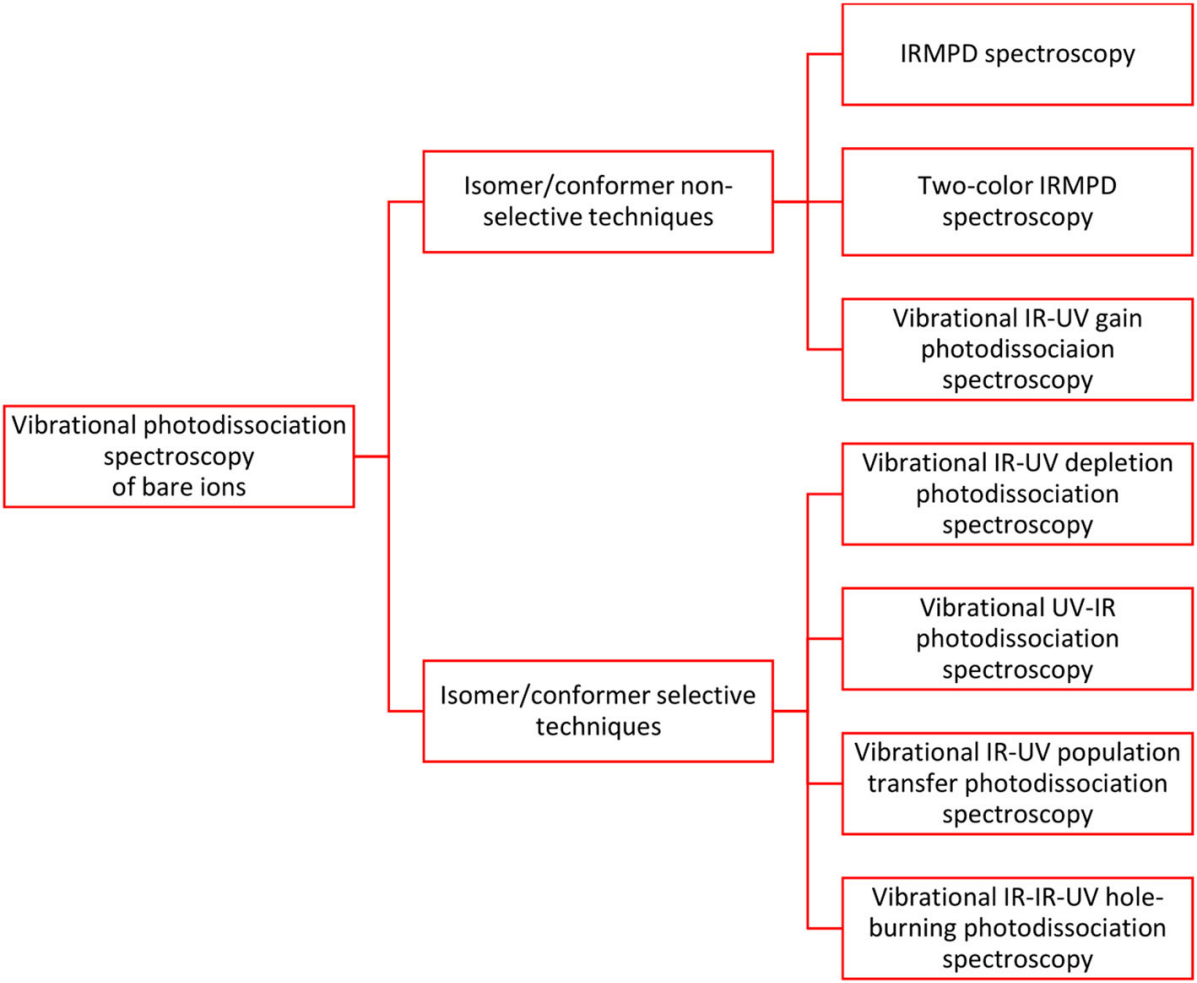
Classification of methods for vibrational photodissociation spectroscopy of bare ions

Scheme 2 summarizes the spectroscopic methods used to obtain vibrational photodissociation spectra of bare ions. In the following, we provide brief details about each of these methods. The most frequent terms used in this field are summarized in Table 3. The full list of terms can be found in Table S3.

**TABLE 3:** Terms for vibrational photodissociation spectroscopy of bare ions (see Table S3 for the complete list)

Name of a technique	Other terms used in literature
**Isomer/conformer non‐selective techniques**	
**Infrared multiple photon dissociation (IRMPD) spectroscopy**	Infrared multiple photon dissociation (IRMPD) spectroscopy IRMPD action spectroscopy Gas‐phase IRMPD spectroscopy Resonant IRMPD (R‐IRMPD) spectroscopy Infrared multiphoton dissociation spectroscopy
**Two‐color infrared multiple photon dissociation (IRMPD) spectroscopy**	Resonance‐enhanced two‐laser IRMPD spectroscopy Two‐color IR dissociation spectroscopy Two‐laser photodissociation spectroscopy Infrared laser‐assisted photodissociation spectroscopy (IRLAPS) Two‐laser infrared laser‐assisted photodissociation spectroscopy (IRLAPS)
**Vibrational IR–UV gain photodissociation** ion **spectroscopy**	IR–UV gain spectroscopy Conformer nonselective vibrational spectroscopy Gain spectroscopy Infrared fragment ion‐gain spectroscopy (IRFIGS) IR–UV photofragment spectroscopy
**Isomer/conformer selective techniques**	
**Vibrational IR–UV depletion photodissociation** ion **spectroscopy**	IR–UV depletion spectroscopy Conformer‐selective IR–UV depletion spectroscopy Conformer‐selective IR–UV double‐resonance photofragment spectroscopy IR–UV double‐resonance spectroscopy Infrared fragment ion‐dip spectroscopy (IRFIDS)
**Vibrational IR–UV population transfer photodissociation** ion **spectroscopy**	IR‐induced population transfer spectroscopy IR population transfer spectroscopy
**Vibrational IR–IR–UV hole‐burning photodissociation** ion **spectroscopy**	IR–IR–UV hole‐burning spectroscopy IR–IR–UV hole‐burning vibrational spectroscopy IR–IR–UV hole‐burning photofragmentation spectroscopy Conformer‐selective IR cold ion spectroscopy (CIS)

### 4.1 Infrared multiple photon dissociation (IRMPD) spectroscopy

For the majority of ions, the energy of a single IR photon is insufficient to induce a dissociation. However, using a high‐power light sources, typically CO_2_ lasers or FEL, enables absorption of multiple photons.^23^, ^24^ The combined energy is then sufficient to overcome the dissociation energy limit. CO_2_ lasers are not tunable in a wide range and thus cannot be used for spectral measurements. FEL, on the other hand, are tunable in a wide range and are accessible as multiuser facilities to many users.^5^, ^25^, ^26^, ^27^ Therefore, infrared multiple photon dissociation spectroscopy has been used to study many different ions. This technique is often also referred to as infrared multiphoton dissociation spectroscopy. Both naming variants share the IRMPD abbreviation.

### 4.2 Two‐color infrared multiple photon dissociation (IRMPD) spectroscopy

Dissociation of bare ions requires absorption of multiple IR photons. However, the initial resonant absorption of one or two photons can be decoupled from the IRMPD process, if one uses two lasers.^28^ The first tunable IR laser induces a resonant absorption and thus makes the photodissociation wavelength dependent. The second laser (typically CO_2_) is used for infrared laser assisted photodissociation (IRLAPS). This method is more experimentally demanding than simple IRMPD spectroscopy, but it has an advantage over IRMPD because it lacks power broadening and redshifts of the spectral lines typically present in IRMPD spectra measured with high‐power laser sources.^29^ As was mentioned before, the IRLAPS might also be used to enhance UV/Vis photodissociation. We thus suggest using the term **two‐color infrared multiple photon dissociation (IRMPD) spectroscopy** to avoid confusion.

### 4.3 Vibrational IR–UV gain photodissociation ion spectroscopy

Cooling of ions to cryogenic temperatures confines the ions in their ground vibrational state and reduces thermal broadening of the electronic transitions. Absorption of an IR photon by any isomer/conformer leads to its internal heating and, thus, to a broadening and to a redshift of the corresponding electronic spectrum. These changes in the electronic absorption are used to record infrared photodissociation spectra of ions. In vibrational IR–UV gain spectroscopy, the UV laser wavelength is tuned to the red of the lowest energy electronic transition of the cold ions and thus does not result in dissociation. The spectral broadening induced by IR photon absorption increases dissociation induced by the following UV photon absorption (signal gain), which is recorded as a function of the IR laser wavelength.^30^

### 4.4 Vibrational IR–UV depletion photodissociation ion spectroscopy

In IR–UV depletion spectroscopy, the UV laser is fixed at the electronic transition specific to the selected isomer/conformer. The absorption of a preceding IR photon by the same isomer/conformer results in the depopulation of the ground vibrational state and in the broadening the electronic spectrum. These factors decrease the dissociation yield induced by the absorption of a UV photon by the ions at the given fixed wavelength.^31^ Vibrational spectra are recorded by monitoring the UV dissociation signal depletion as a function of the IR laser wavelength. This technique is sensitive to different isomers and conformers, which also often surfaces in the used name of spectroscopy (isomer‐specific/selective, conformer‐specific/selective). This double‐resonance technique requires a well‐resolved electronic transitions, which means that the ions have to be cooled to their ground vibrational state. The necessity of cooling to a low temperature during experiments is also often emphasized in the name of the given spectroscopy (see Table S3 for all variants).

### 4.5 Vibrational UV–IR photodissociation ion spectroscopy (IR spectra of ions in electronically excited states)

The order of the IR and UV absorption can be reversed (UV–IR spectroscopy). The first UV laser wavelength is tuned to a specific electronic excitation band. The subsequent IR photon absorption by the ion in the excited state substantially enhances its dissociation. The delay between the laser pulses must be as short as possible, limited by the lifetime of the exited state and the pulse width (typically, a few ns). The increase in the dissociation yield is recorded as a function of the IR wavelength, allowing for the measurement of IR spectra of the ions in the excited states.^32^ The fact that one probes a specific electronic absorption makes this technique isomer/conformer specific.

### 4.6 Vibrational IR–UV population transfer photodissociation ion spectroscopy

Another example of a double resonance experiment is an IR variant of the IR–UV population transfer experiment. The UV laser is fixed at a transition belonging to a particular isomer/conformer. IR excitation is timed to precede the UV laser, giving sufficient time for the vibrationally excited ions to isomerize and to cool by collisions with neutral molecules prior to the UV excitation (typically, several milliseconds). In this scheme, depletion of a photodissociation signal results from an isomerization of the UV probed conformer to another accessible conformation.^22^ The photodissociation signal can also gain intensity by other conformations isomerizing to the conformer interrogated by the UV laser.

### 4.7 Vibrational IR–IR–UV hole‐burning photodissociation ion spectroscopy

The isomer/conformer selective vibrational photodissociation spectroscopy methods described above can only be applied to the ions that have well‐resolved isomer‐specific electronic transitions in the ground vibrational state. The IR–IR–UV hole‐burning technique overcomes this limitation by using an additional IR laser to label vibrational rather than electronic isomer/conformer‐specific transitions.^33^ The first pump IR laser selectively saturates the transition of only one conformer, whereas the scanning probe IR laser followed by UV photodissociation probe vibrational IR–UV gain spectroscopy of all the other conformers. The difference of spectra obtained with and without the pump IR laser corresponds to the isomer/conformer specific spectrum of the labeled conformer. The term hole‐burning refers to the disappearance of the vibrational transitions that belong to the labeled conformer from the IR–UV gain spectrum.

## 5 CLASSIFICATION OF METHODS OF THE PHOTODISSOCIATION SPECTROSCOPY OF IONS TAGGED BY LIGHT ATOMS OR MOLECULES

**Tagging photodissociation ion spectroscopy** works with ions having very low internal energy. This can be achieved in a supersonic expansion, in a cryogenic ion trap, or by other techniques. The cold ions form non‐covalent complexes with neutral atoms or molecules (He, Ne, Ar, H_2_, H_2_O, etc.), referred to as “adduct,” “messenger,” “molecular messenger,” “mass messenger,” “messenger tag,” or simply “tag.” The assumption of the tagging method is that the binding of the tag does not significantly affect the structure of the original bare ion. Accordingly, the spectra obtained by tagging spectroscopy can be related to the structure of the “bare” ions. If the interaction between a studied ion and a non‐covalently bound molecule affects the structure of the ion, the cluster should be considered as a new system and must not be associated with the tagging photodissociation ion spectroscopy.

Absorption of a photon results in the dissociation of these weakly bound complexes. This process is referred to as “evaporation,” or “photo‐evaporation.” Another term is “predissociation,” which refers to the fact that the photon absorption populates a quazibound (predissociated) state and subsequently the complex dissociates. For the sake of consistency, we suggest using the simple term **dissociation**. Researchers refer to these techniques by specifying the tag and/or they often emphasize the necessity of low temperature by using adjectives such as cryogenic or cold‐ion (see Tables 4 and S4).

**TABLE 4:** Terms for tagging photodissociation ion spectroscopy (see Table S4 for the complete list)

Name of a technique	Other terms used in literature
**Tagging photodissociation** ion **spectroscopy**	Tagging spectroscopy Messenger spectroscopy Cryogenic messenger‐tagging spectroscopy Tagging action spectroscopy Predissociation spectroscopy
**Electronic (UV/Vis) tagging photodissociation** ion **spectroscopy**	Tagging Vis spectroscopy Tagging photodissociation spectroscopy Tagging predissociation spectroscopy UV tagging spectroscopy Vis photodissociation (VisPD) spectroscopy
**Vibrational tagging photodissociation** ion **spectroscopy**	Tagging IR photodissociation (IRPD) spectroscopy Messenger‐tagging IR spectroscopy IR predissociation spectroscopy Vibrational predissociation spectroscopy Cryogenic ion vibrational predissociation (CIVP) spectroscopy
**Vibrational light‐induced inhibition of complex growth (LIICG)** ion **spectroscopy**	Vibrational spectroscopy
**Vibrational tagging IR–IR hole burning photodissociation** ion **spectroscopy**	IR–IR hole‐burning spectroscopy IR–IR double‐resonance messenger‐tagging spectroscopy Isomer‐selective messenger tagging spectroscopy Cryogenic IR–IR double‐resonance spectroscopy Two‐color IR–IR photodissociation spectroscopy

**TABLE 5:** Terms for rotational action ion spectroscopy (see Table S6 for the complete list)

Suggested terms	Other terms used in literature
**Laser‐induced reaction (LIR) ion spectroscopy**	
**Rotational laser‐induced reaction (LIR)** ion **spectroscopy**	Pure rotational spectroscopy Rotational spectroscopy
**Rotationally resolved vibrational laser‐induced reaction (LIR)** ion **spectroscopy**	High‐resolution IR spectroscopy
**IR‐THz laser‐induced reaction (LIR)** ion **spectroscopy**	Pure rotational spectroscopy IR‐THz double‐resonance LIR depletion spectroscopy
**Tagging photodissociation ion spectroscopy**	
**Rotational laser‐induced inhibition of complex growth (LIICG)** ion **spectroscopy**	Rotational spectroscopy
**THz‐IR tagging photodissociation** ion **spectroscopy**	Double‐resonance rotational spectroscopy

**SCHEME 3:**
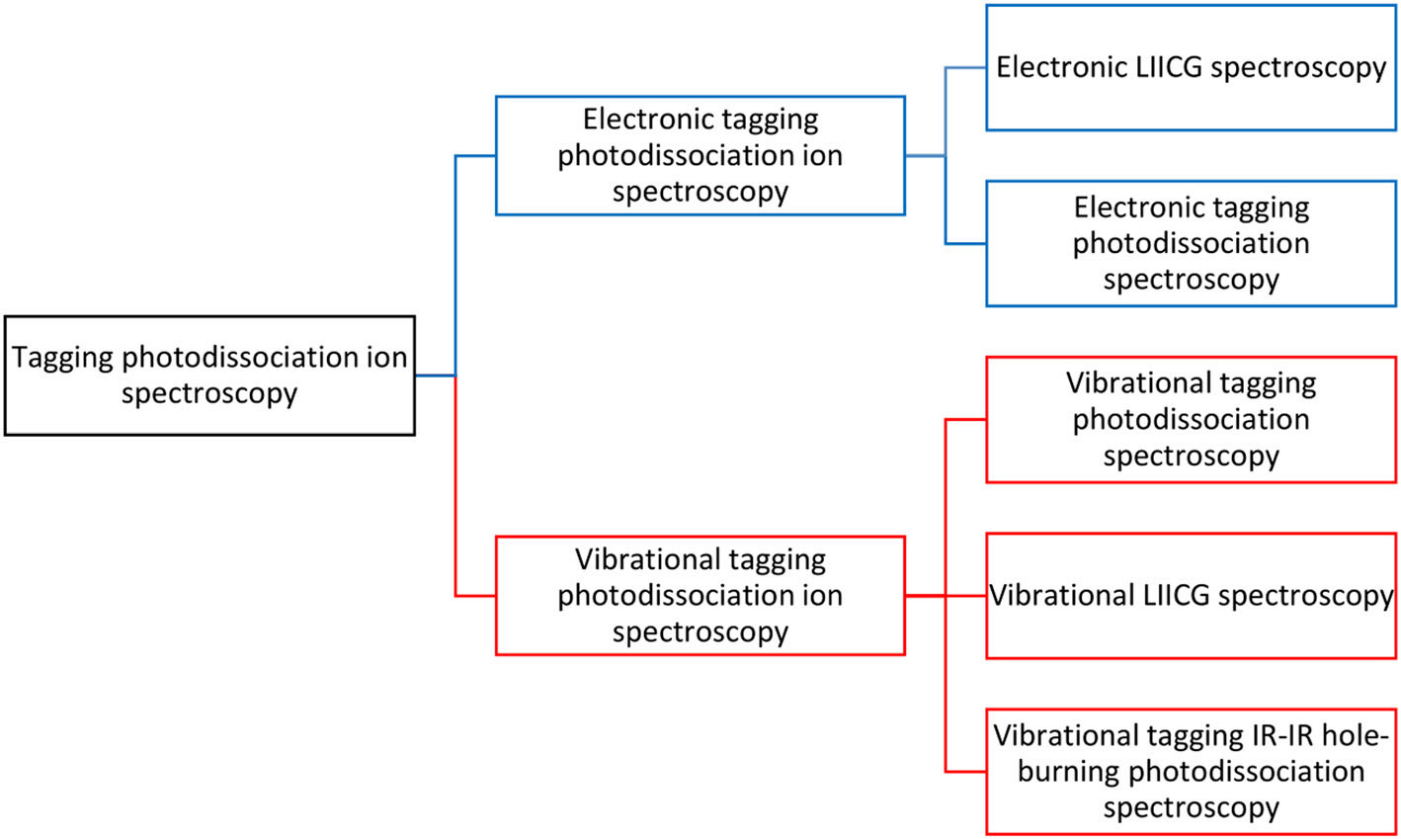
Classification of the methods for electronic and vibrational tagging photodissociation ion spectroscopy of ions tagged by light atoms/molecules

In contrast to photodissociation spectroscopy of bare ions where different laser pulses have a unique effect, the tagging photodissociation spectroscopy is much more straightforward in the sense that absorption of any photon, either UV or IR, leads to the dissociation of the weakly bound complex, which makes one‐color experiments isomer/conformer nonselective. When more than one isomers/conformers are present, any of the reported techniques can be extended to a conformer‐selective method by adding a second laser to perform hole‐burning experiments. Next to the tagging IR–IR hole‐burning experiments, one can imagine tagging IR–UV or UV–UV, or even UV–IR hole burning techniques, which, however, have not been reported so far. Scheme 3 summarizes the reported spectroscopic methods used to obtain photodissociation spectra of ions tagged by light atoms/molecules. In the following, we provide brief details about all these methods. The terms used in the field are summarized in Table 4 (for the full list of terms, see Table S4).

### 5.1 Electronic light‐induced inhibition of complex growth (LIICG) ion spectroscopy

Despite the low binding energy of noble gas tags, their presence might significantly alter their electronic^34^ and even vibrational^35^ spectra of small ions. Therefore, related techniques working with bare ions were developed. These techniques are based on the reactivity differences between the ions in their ground states and the excited states. Laser‐induced inhibition of complex growth (LIICG) spectroscopy relies on a decreased ability to form the tagged complexes while the ions are in the excited state. Hence, one monitors wavelength‐dependent inhibition of the growth of the tagged ions upon simultaneous photon absorption with a continuous source.^36^

### 5.2 Electronic (UV/Vis) tagging photodissociation ion spectroscopy

Tagging photodissociation spectroscopy detects dissociation of the complexes of ions with neutral tags formed before the irradiation. The weakly bound ion–neutral complexes are irradiated by a laser light in the UV or visible range. Following the electronic excitation, these complexes lose the weakly bound tag, which results in the parent ion signal depletion. The spectra are recorded by monitoring depletion of the signal as a function of laser wavelength.^37^

### 5.3 Vibrational tagging photodissociation ion spectroscopy

The main advantage of tagging photodissociation spectroscopy is that the energy required to dissociate the loosely bound complex is small compared with the dissociation energy of covalent bonds. Absorption of one IR photon is usually sufficient to excite the complex to a quazibound (predissociated) state, which is followed by the elimination of the tag. The spectra are recorded as a wavelength‐dependent signal depletion of the tagged complexes.^38^

### 5.4 Vibrational light‐induced inhibition of complex growth (LIICG) ion spectroscopy

The principle of the electronic LIICG spectroscopy can be applied also for vibrational excitation. Absorption of the IR photon heats up the ion, decreasing its efficiency of the complexation with neutral tag molecules.^39^

### 5.5 Vibrational tagging IR–IR hole‐burning photodissociation ion spectroscopy

When two or more isomers/conformers with distinct IR spectra are present in the sample, an additional IR pulse can be used to selectively dissociate one of them. The pump laser is fixed to excite aspecific vibrational transition of a given isomer/conformer, leading to its dissociation. The following scanning probe IR laser records the spectrum containing transitions of all other isomers. The conformer‐specific spectrum is obtained as a difference of the spectra recorded with and without pump IR irradiation.^40^

## 6 OTHER METHODS FOR GAS‐PHASE ION SPECTROSCOPY

Photodissociation of bare ions or weakly bound complexes are the most widespread, but not the only methods for recording spectra of ions in the gas phase. In the following, we describe alternative physical principles of signal detection in ion spectroscopy.

### 6.1 Electronic gas‐phase luminescence ion spectroscopy

Although the direct absorption spectroscopy of mass selected ions trapped in the rf trap is impossible due to low ion concentrations, their luminescence at wavelength different from the excitation can be measured directly because of a “zero” background.^41^ The experiments are usually performed in dedicated Paul‐type trap instruments. The terminology is the same as in classical luminescence spectroscopy with using adjective “gas‐phase” to stress the type of the technique.

### 6.2 Vibrational laser‐induced reaction (LIR) ion spectroscopy

Spectroscopic investigation of small ions composed of only a few atoms is extremely complicated. The electronic excitations are often lying in the VUV spectral range, whereas the available excitation ranges are limited to IR or THz radiation. Furthermore, due to the small size of the ions, their tagging with neutral atoms is extremely inefficient. Therefore, an alternative, laser‐induced reaction (LIR) spectroscopy, has been developed. This method makes use of an opening of a reaction pathway in a vibrationally excited state. Hence, the “action” is a reaction of the excited ions. The yield of the reaction products thus depends of the excitation wavelength and reflects the IR spectra of the studied ions.^42^ This method has been successfully employed to obtain vibrational, rovibrational, and pure rotational spectra of many ions. In order to differentiate between these LIR methods, we suggest using it with the term explicitly specifying the spectroscopic outcome. The LIR method for obtaining IR spectra of ions should be referred to as **vibrational LIR spectroscopy**.^43^ Applications of this method for obtaining information regarding rotational transitions of ions will be discussed in the next section.

### 6.3 Rydberg photoionization ion spectroscopy

Typical ionization threshold energy of small molecules and ions is larger than the photon energies of most laser sources. However, it can be possible to use intermediate electronically excited states to overcome this ionization threshold energy. Low‐lying Rydberg states can be used as such intermediates giving rise to numerous specific spectroscopic techniques such as zero electron kinetic energy (ZEKE) spectroscopy and mass‐analyzed threshold ionization (MATI) spectroscopy, as well as their derivatives.^44^, ^45^ These techniques lie beyond the scope of the current review.

### 6.4 Direct absorption spectroscopy

As was mentioned earlier, gas‐phase action ion spectroscopy was developed to overcome the problem of the very low concentration of ions in ion traps that typically do not allow direct absorption spectroscopy. However, on the dawn of ion spectroscopy, several direct absorption experiments have been demonstrated using discharge cells with relatively high gas concentration ( $\sim 10^{11}\;{\mathrm{cm}}^{-3}$) ionized by a discharge. Such approach allowed researchers to measure direct electronic absorption (cavity ring‐down spectroscopy)^46^ and to apply infrared absorption spectroscopy^47^ and microwave absorption spectroscopy^48^ for small ions. These techniques lie beyond the scope of the current review of techniques for mass‐selected ions.

## 7 CLASSIFICATION OF METHODS OF ROTATIONAL ACTION ION SPECTROSCOPY

**SCHEME 4:**
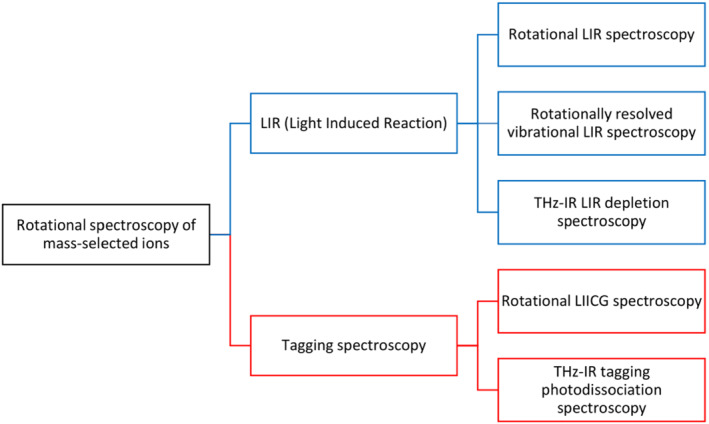
Classification of methods for rotational action ion spectroscopy

Rotational spectroscopy is limited to only small ions composed of several atoms. This type of spectroscopy is the most challenging one because of a small size of the ions of interest within this technique. All the developed spectroscopic methods rely on two detection schemes—laser‐induced reactivity (LIR) and tagging spectroscopy. Scheme 4 summarizes the reported spectroscopic methods used to obtain rotational and rovibrational spectra of mass‐selected ions. In the following, we provide details about all these methods. The terms used in the field are summarized in Table 4.

### 7.1 Rotational laser‐induced reaction (LIR) ion spectroscopy

This method relies on endothermic reactions of cold ions with a gas in an ion trap. The reactions do not occur unless the ions get internally excited. The pure rotational excitations can be studied with narrowband tunable light sources operating in the THz frequency region. The rotational spectra of the ions are measured by monitoring the number of the laser‐induced reaction product ions as a function of the excitation frequency.^49^

### 7.2 Rotationally resolved vibrational laser‐induced reaction (LIR) ion spectroscopy

Another method for studying rotational transitions uses a high‐resolution IR source, such as an OPO. Excitation of rovibrational transitions can promote reactivity which is observed as an increased product yield. This method is preferred when the reaction energy barrier is high.^42^

### 7.3 THz‐IR laser‐induced reaction (LIR) ion spectroscopy

In these experiments, ions are irradiated simultaneously with IR and THz radiations. The IR laser is tuned to a rovibrational transition starting either at the ground state or at the excited rotational state and promotes reactivity, which is monitored as a number of the product ions. The reaction output can be modulated with THz radiation. If the rotational excitation depopulates the initial state probed by IR laser, the reactivity decreases, whereas if the rotational excitation populates the initial state probed by an IR laser, the reactivity increases.^50^

### 7.4 THz‐IR tagging photodissociation ion spectroscopy

This technique is almost identical to the previous one except that the spectra are recorded by monitoring the number of He‐tagged ions, rather than the light‐induced reaction products. The infrared laser is stabilized at a transition that leads to the dissociation of weakly bound complexes. THz radiation either populates or depopulates rotational state probed by the IR radiation, leading to the gain or depletion of the signal.^51^

### 7.5 Rotational laser‐induced inhibition of complex growth (LIICG) ion spectroscopy

Rotational LIICG spectroscopy is a spectroscopic technique that exploits the dependence of the ternary attachment of neutral atoms (typically He) on the rotational quantum state. The ions are irradiated by mm‐wavelength light and then reacted with He. The dips in the numbers of the formed He complexes as a function of the wavelength indicate the rotational excitations of the bare ions.^52^

## 8 POSSIBLE EXTENSIONS AND APPLICATIONS OF THE SPECTROSCOPIC METHODS

The availability of ion spectroscopy in many laboratories opens many possibilities for coupling the experiments with other techniques. These techniques can involve various ways of the preparation of the studied ions and can also involve various ways of the manipulation of the ions prior to their spectroscopic interrogation. The coupling of the techniques can lead to unique results revealing the properties of the ions. However, the coupled techniques do not affect the art of ion spectroscopy. Therefore, they should be mentioned separately and should not become a part of the name of the spectroscopic method. Therefore, they are not addressed here other than a representative example.

### 8.1 Action ion spectroscopy of mobility‐selected ions

Ion mobility is an example of a valuable extension of techniques used together with ion spectroscopy methods. For example, one can separate anomers of ionized sugars by ion mobility and afterward investigate them separately by electronic and vibrational photodissociation spectroscopy.^53^, ^54^ Such an extension can be coupled with any of the methods listed previously. The ion mobility separation of the ions does not affect the spectroscopic technique; therefore, “ion mobility” should not appear in the naming of the spectroscopic techniques, but should appear separately.

### 8.2 Gas‐phase FRET studied by electronic photodissociation and fluorescence ion spectroscopies

Förster resonance energy transfer (FRET) process between two chromophores of an ion can be studied in the gas phase. Fluorescence of mass‐selected ions as well as FRET can be studied in ion traps directly by detecting photons (see luminescence spectroscopy, part 5).^55^ Within photodissociation spectroscopy, FRET can be studied too. It is possible, if the dissociation pattern of the electronically excited ions changes after the Förster resonance energy transfer. This technique is often referred to as action FRET. In fact, it is a classical FRET detected by photodissociation electronic spectroscopy that was performed on chemically modified^56^ and, later, unmodified^57^ ions in the gas phase.

## 9 NAMES USED TO DESCRIBE SPECTRA OF IONS

### 9.1 The names of the spectra obtained by gas‐phase action ion spectroscopy methods

The spectra of ions recorded in the gas phase differ fundamentally from the ones recorded in solution. Furthermore, the experimental methods to obtain the spectra are very diverse (see all the methods earlier) and may affect the intensities of the bands in the spectra, resolution, and sometimes even the band positions. Therefore, the methods must be always described along with the published spectra, and the name of the spectrum should, ideally, include the name of the experimental method. We list a rather long list of the collected spectra names in the Supporting Information (Table S7) together with the relevant citations. Obviously, many more variations exist in literature. This only highlights the necessity for a clear description of the spectra.

## 10 CONCLUSIONS

The presented classification of the action ion spectroscopic techniques best illustrates different approaches for characterization of mass‐selected ions in the gas phase. The names of the techniques proposed herein (except for only a few specific exceptions) specify the nature of the probed spectroscopic transition, experimental scheme, and the detection principle. This allows for an unambiguous description of each method. Without a doubt, each invention and each push toward a better resolution, a better selectivity, or a better sensitivity deserves an applause. However, such developments do not need to be accompanied with new names or new abbreviations for the particular spectroscopic method. Our efforts should go toward making action ion spectroscopy easy to grasp by scientists outside of this field. Simple, easy‐to‐understand, and easy‐to‐remember terms would help in this effort.

## Supporting information


**Table S1.** General terms for action ion spectroscopy in the gas phase
**Table S2.** Terms for electronic photodissociation spectroscopy of bare ions
**Table S3.** Terms for vibrational photodissociation spectroscopy of bare ions
**Table S4.** Terms for photodissociation spectroscopy of ions tagged by light atoms/molecules.
**Table S5.** Terms for other methods in gas‐phase action ion spectroscopy
**Table S6.** Terms for rotational action ion spectroscopy
**Table S7.** Notation in names of spectra
