# Supplementary material for: Experimental techniques and terminology in gas‐phase ion spectroscopy
Source: J Mass Spectrom. 2022 Apr 17;57(5):e4826. doi: 10.1002/jms.4826 (PMC9285946; doi:10.1002/jms.4826)
Supplement: Supplementary file 1 — Table S1. General terms for action ion spectroscopy in the gas phase Table S2. Terms for electronic photodissociation spectroscopy of bare ions Table S3. Terms for vibrational photodissociation spectroscopy of bare ions Table S4. Terms for photodissociation spectroscopy of ions tagged by light atoms/molecules. Table S5. Terms for other methods in gas‐phase action ion spectroscopy Table S6. Terms for rotational action ion spectroscopy Table S7. Notation in names of spectra [file JMS-57-0-s001.pdf]

# Supporting Information for

# Experimental techniques in gas-phase action ion spectroscopy

Aleksandr Pereverzev and Jana Roithová

*Institute for Molecules and Materials, Radboud University, Heyendaalseweg 135, 6525 AJ Nijmegen, The Netherlands, E-mail: j.roithova@science.ru.nl.*

## Table of Contents

Table S1. General terms for action ion spectroscopy in the gas phase ..... 2

Table S2. Terms for electronic photodissociation spectroscopy of bare ions ..... 5

Table S3. Terms for vibrational photodissociation spectroscopy of bare ions ..... 7

Table S4. Terms for photodissociation spectroscopy of ions tagged by light atoms/molecules ..... 9

Table S5. Terms for other methods in gas-phase action ion spectroscopy ..... 11

Table S6. Terms for rotational action ion spectroscopy ..... 12

Table S7. Notation in names of spectra ..... 13

Bibliography ..... 21

**Table S1. General terms for action ion spectroscopy in the gas phase**

| Name of the technique | Other terms used in literature |
|---|---|
| **Gas-phase action ion spectroscopy** | Ion spectroscopy [1–20]<br>Gas-phase spectroscopy [8,13,21–29]<br>Action spectroscopy [4,8,9,12–15,18,19,24,26,29–42]<br>Consequence spectroscopy [14]<br>Ion trapping spectroscopy [41]<br>Gas-phase action spectroscopy [3,39]<br>Gas-phase ion spectroscopy [26,42,43]<br>Laser spectroscopy [1,14,18,23,44–47]<br>Optical spectroscopy [1,2,34,38,48]<br>Absorption spectroscopy [26,39]<br>Optical absorption spectroscopy [2]<br>Gas-phase absorption spectroscopy [26,39]<br>Gas-phase ion absorption spectroscopy [26]<br>Absorption action spectroscopy [39]<br>Mass-selective action spectroscopy [49] |
| **Gas-phase photodissociation ion spectroscopy** | Photodissociation spectroscopy [1,11,13,45,48,50–53]<br>Photofragment spectroscopy [23,54–59]<br>Photofragmentation spectroscopy [60,61]<br>Photodissociation action spectroscopy [42]<br>Action photodissociation spectroscopy [51]<br>Resonance-enhanced photodissociation spectroscopy [4]<br>Laser dissociation spectroscopy [62]<br>Photofragmentation cold ion spectroscopy (CIS) [27] |
| **Cryogenic gas-phase action ion spectroscopy (CIS)** | Cryogenic ion spectroscopy (CIS) [63]<br>Cold ion spectroscopy (CIS) [5,6,11,14,21,27,28,55,59–61,64–69]<br>Gas-phase cold ion spectroscopy (CIS) [21]<br>Cryo-cooled ion spectroscopy [61]<br>Cryo-cooled gas-phase spectroscopy [43] |
| **Electronic gas-phase action ion spectroscopy** | Electronic spectroscopy [3,9,13,37,55,57,70–72]<br>Electronic ion spectroscopy [9]<br>UV spectroscopy [3,6,25,28,43,46,47,68,73]<br>UV ion spectroscopy [9]<br>UV cold ion spectroscopy (CIS) [68]<br>UV photodissociation (UVPD) spectroscopy [4]<br>UV photofragment spectroscopy [43] |

| | |
|---|---|
| | UV/Vis spectroscopy [4,7,52,74]<br>UV/Vis ion spectroscopy [13]<br>UV/Vis absorption spectroscopy [75]<br>Vis spectroscopy [74,76]<br>Vis laser spectroscopy [74]<br>Gas-phase electronic spectroscopy [3,9]<br>Gas-phase UV spectroscopy [3]<br>Gas-phase UV/Vis spectroscopy [3]<br>Cold ion UV spectroscopy [77]<br>UV cold ion spectroscopy (CIS) [77] |
| **Vibrational gas-phase action ion spectroscopy** | Vibrational spectroscopy [2,13,14,24,33,55,58,63–65,70,72,78–86]<br>IR spectroscopy [2,3,6–9,13–15,23–25,28,32,36–38,43,46,48,51,54,64,68,73–77,81,87–100]<br>Mid-IR spectroscopy [13,51]<br>IR vibrational spectroscopy [41]<br>IR ion spectroscopy [8,9,14,18,19]<br>Gas-phase vibrational spectroscopy [3,78,80]<br>Gas-phase IR spectroscopy [8,43,51,89,101]<br>Gas-phase mid-IR spectroscopy [51]<br>Vibrational action spectroscopy [35,78,80]<br>IR action spectroscopy [14,41]<br>IR photodissociation (IRPD) spectroscopy [4,18,37,48,53]<br>Vibrational photofragment spectroscopy [58]<br>Vibrational cold ion spectroscopy (CIS) [99]<br>Cold ion IR spectroscopy [66]<br>IR cold ion spectroscopy (CIS) [27,69]<br>Cryogenic ion trap vibrational spectroscopy [78]<br>Cryogenic vibrational spectroscopy [89,102]<br>Cryogenic IR spectroscopy [98,102,103] |
| **Rotational gas-phase action ion spectroscopy** | Rotational spectroscopy [33,35,88]<br>Rotational action spectroscopy [35]<br>Microwave spectroscopy [91] |
| Multi-resonance spectroscopic techniques | Double-resonance spectroscopy [60,79,96,104]<br>Double-resonance ion spectroscopy [4]<br>Double-resonance laser spectroscopy [67]<br>IR-UV spectroscopy [4]<br>IR-UV photofragment spectroscopy [54,102]<br>IR-UV photodissociation spectroscopy [27]<br>IR-UV photofragmentation vibrational spectroscopy [99]<br>Double-resonance IR-UV spectroscopy [4] |

| | |
|---|---|
| | IR-UV double-resonance spectroscopy [5,13,22,25,28,43,44,46,67,73,96,97,104,105]<br>UV-IR double-resonance spectroscopy [47]<br>IR-UV double-resonance vibrational spectroscopy [4]<br>IR-UV double-resonance photofragment spectroscopy [6,43,56]<br>IR-UV double-resonance photofragmentation spectroscopy [79,104]<br>IR-UV double-resonance cold ion spectroscopy (CIS) [28,65] |
| Isomer/conformer selectivity | Conformation-specific spectroscopy [22,59,60]<br>Conformationally specific spectroscopy [40]<br>Single conformation spectroscopy [67]<br>Isomer-selective spectroscopy [72]<br>Conformer-selective cold ion spectroscopy (CIS) [28]<br>Conformation-selective electronic spectroscopy [44]<br>Conformation-specific UV spectroscopy [61]<br>Conformer-selective vibrational spectroscopy [79]<br>Conformer-selective IR spectroscopy [66,79]<br>IR conformer-selective spectroscopy [27]<br>Conformer-specific IR spectroscopy [79]<br>Conformation-specific IR spectroscopy [61]<br>Conformer-selective cryogenic IR spectroscopy [63] |

**Table S2. Terms for electronic photodissociation spectroscopy of bare ions**

| Name of the technique | Other terms used in literature |
|---|---|
| **Isomer/conformer non-selective techniques** | |
| **Electronic (UV/Vis) photodissociation ion spectroscopy** | Electronic spectroscopy [55]<br>UV spectroscopy [3,73]<br>UV action spectroscopy [9]<br>UV/Vis electronic spectroscopy [13]<br>UV/Vis action spectroscopy [13]<br>Gas-phase UV spectroscopy [86]<br>Electronic photodissociation spectroscopy [37]<br>UV photodissociation (UVPD) spectroscopy [4,25]<br>UV photofragment spectroscopy [22,54,56,59,61,79,97]<br>UV photofragmentation spectroscopy [47,86,97,105]<br>Laser-induced UV photofragmentation spectroscopy [43,59]<br>Photodissociation spectroscopy [1,106]<br>Photofragment spectroscopy [55]<br>Photo-induced dissociation action spectroscopy [26] |
| **Electronic infrared laser-assisted photodissociation ion spectroscopy (IRLAPS)** | Infrared laser-assisted photofragment spectroscopy [23,56]<br>Two-laser multiphoton dissociation spectroscopy [107] |
| **2D UV-MS photodissociation ion spectroscopy** | 2D UV–MS spectroscopy [77,108]<br>2D UV–MS cold ion spectroscopy (CIS) [77,109]<br>UV fragmentation spectroscopy–mass spectrometry (UV–MS) [66] |
| **Circular dichroism ion spectroscopy** | Electronic circular dichroism (CD) ion spectroscopy [19]<br>Circular dichroism (CD) spectroscopy [19] |
| **Isomer/conformer selective techniques** | |
| **Electronic UV-UV hole-burning photodissociation ion spectroscopy** | UV-UV hole-burning spectroscopy [25,44,60,110]<br>UV-UV double-resonance spectroscopy [60] |
| **Electronic IR-UV hole-burning photodissociation ion spectroscopy** | IR-UV hole-burning spectroscopy [28,44,60] |

| | |
|---|---|
| **Electronic IR-UV population transfer photodissociation ion spectroscopy** | IR-UV hole-filling spectroscopy [79]<br>IR-UV population transfer hole-filling spectroscopy [61] |

**Table S3. Terms for vibrational photodissociation spectroscopy of bare ions**

| Name of a technique | Other terms used in literature |
|---|---|
| **Isomer/conformer non-selective techniques** | |
| **Infrared multiple photon dissociation (IRMPD) spectroscopy** | Infrared multiple photon dissociation (IRMPD) spectroscopy [8,14,27,32,36,51,55,58,59,78,83,89,92,99,103]<br>IRMPD action spectroscopy [83]<br>Mid-IRMPD spectroscopy [92]<br>Gas-phase IRMPD spectroscopy [32,51]<br>Resonant IRMPD (R-IRMPD) spectroscopy [90]<br>Resonance-enhanced IRMPD spectroscopy [37]<br>Infrared multiphoton dissociation spectroscopy [4,7,9,13,20,23,48,80,101,105,111]<br>Infrared multiphoton dissociation action spectroscopy [9]<br>Resonant infrared multiphoton dissociation spectroscopy [91]<br>IR multiphoton spectroscopy [20]<br>IR photodissociation (IRPD) spectroscopy [48,78,91]<br>Gas-phase mid-IR photodissociation spectroscopy [101]<br>Vibrational spectroscopy [18]<br>Mid-IR spectroscopy [32] |
| **Two-color infrared multiple photon dissociation (IRMPD) spectroscopy** | Resonance-enhanced two-laser IRMPD spectroscopy [75]<br>Two-color IR dissociation spectroscopy [112]<br>Two-laser photodissociation spectroscopy [75]<br>Infrared-laser assisted photodissociation spectroscopy (IRLAPS) [58]<br>Two-laser infrared laser-assisted photodissociation spectroscopy (IRLAPS) [58] |
| **Vibrational IR-UV gain photodissociation ion spectroscopy** | IR-UV gain spectroscopy [99]<br>Conformer nonselective vibrational spectroscopy [27]<br>Gain spectroscopy [4]<br>IR photofragment gain spectroscopy [6]<br>Infrared fragment ion-gain spectroscopy (IRFIGS) [6,97]<br>IR-UV photofragment spectroscopy [56] |
| **Isomer/conformer selective techniques** | |
| **Vibrational IR-UV depletion photodissociation ion spectroscopy** | IR-UV depletion spectroscopy [6,28,67,77,104]<br>IR-UV photofragmentation depletion spectroscopy [104]<br>Conformer-selective IR-UV depletion spectroscopy [104]<br>Conformer-specific IR depletion spectroscopy [22] |

| | |
|---|---|
| | Conformer-selective cryogenic IR spectroscopy [63]<br>Conformer-selective cold ion IR-UV spectroscopy [21]<br>Conformer-selective IR-UV double resonance spectroscopy [56]<br>Conformation-specific IR-UV double resonance spectroscopy [61,105]<br>Conformer-selective IR-UV double resonance photofragment spectroscopy [59]<br>IR-UV double resonance spectroscopy [59,93,96]<br>IR-UV double resonance vibrational spectroscopy [28]<br>IR-UV double-resonance photofragment spectroscopy [56,59]<br>IR-UV double resonance spectroscopy [13,22,55,73,89,105]<br>Isomer-specific IR spectroscopy [89]<br>IR depletion spectroscopy [23,111]<br>Depletion spectroscopy [79,104]<br>Ion dip spectroscopy [78]<br>Infrared fragment ion-dip spectroscopy (IRFIDS) [97]<br>IR-UV dip spectroscopy [14]<br>IR-UV ion dip spectroscopy [14] |
| **Vibrational IR-UV population transfer photodissociation ion spectroscopy** | IR-induced population transfer spectroscopy [79]<br>IR population transfer spectroscopy [67] |
| **Vibrational IR–IR–UV hole-burning photodissociation ion spectroscopy** | IR–IR–UV hole-burning spectroscopy [79,99]<br>IR-IR-UV hole-burning vibrational spectroscopy [99]<br>IR–IR–UV hole-burning photofragmentation spectroscopy [79]<br>Conformer-selective IR cold ion spectroscopy (CIS) [27] |

**Table S4. Terms for photodissociation spectroscopy of ions tagged by light atoms/molecules.**

| Name of a technique | Other terms used in literature |
|---|---|
| **Tagging photodissociation ion spectroscopy** | Tagging spectroscopy [17,58,70,72]<br>Messenger spectroscopy [6,29,41,43,58]<br>Messenger tagging spectroscopy [85,102]<br>Cryogenic messenger-tagging spectroscopy [8]<br>Tagging action spectroscopy [13]<br>Predissociation spectroscopy [10,48,113]<br>Photo-predissociation spectroscopy [11] |
| **Electronic (UV/Vis) tagging photodissociation ion spectroscopy** | Tagging Vis spectroscopy [17]<br>Tagging photodissociation spectroscopy [74]<br>Tagging predissociation spectroscopy [52]<br>UV tagging spectroscopy [47]<br>Vis photodissociation (VisPD) spectroscopy [52]<br>Vis absorption spectroscopy [74]<br>UV depletion spectroscopy [47]<br>Eectronic spectroscopy [1] |
| **Vibrational tagging photodissociation ion spectroscopy** | Tagging IR photodissociation (IRPD) spectroscopy [7,12,52,53,114]<br>Messenger-tagging IR spectroscopy [102,103]<br>One-photon tagged-ion IR spectroscopy [13]<br>Cluster-ion spectroscopy [30]<br>Messenger spectroscopy [32]<br>IR predissociation spectroscopy [1,41,52,73,111,115]<br>Vibrational predissociation spectroscopy [14,70,89,113,116]<br>IR vibrational predissociation spectroscopy [117]<br>Cryogenic messenger-tagging IR spectroscopy [98]<br>Nonconformer-selective IR tagging spectroscopy [99]<br>Cryogenic vibrational predissociation spectroscopy [40]<br>Cryogenic ion vibrational predissociation (CIVP) spectroscopy [18,61,118]<br>IR photodissociation (IRPD) spectroscopy [1,4,7,30,81,117,119]<br>IR laser photodissociation spectroscopy [1]<br>Mass-selected IR laser photodissociation spectroscopy [1]<br>Vibrational spectroscopy [2,119]<br>IR spectroscopy [2,18,51,64]<br>IR absorption spectroscopy [1]<br>IR dissociation spectroscopy [115]<br>One-photon IR spectroscopy [2] |

| | |
|---|---|
| | Single-photon IR photdissociation (IRPD) spectroscopy [7]<br>Mass-selective photodissociation spectroscopy [1] |
| **Vibrational liht-induced inhibition of complex growth (LIICG) ion spectroscopy** | Vibrational spectroscopy [49] |
| **Vibrational tagging IR-IR hole burning photodissociation ion spectroscopy** | IR-IR hole-burning spectroscopy [44,79,102]<br>IR-IR ion dip spectroscopy [63]<br>IR-IR double resonance spectroscopy [63,102,110]<br>IR-IR double resonance messenger-tagging spectroscopy [102]<br>IR-IR conformation specific spectroscopy [40]<br>Isomer-selective messenger tagging spectroscopy [102]<br>Isomer-specific IR-IR double resonance spectroscopy [84]<br>Conformation-specific vibrational spectroscopy [99]<br>Isomer-specific IR spectroscopy [89]<br>Cryogenic IR-IR double resonance spectroscopy [63]<br>Two-color IR-IR photodissociation spectroscopy [111]<br>Two-color IR photodissociation (IRPD) spectroscopy [7,114]<br>Two-color IR predissociation spectroscopy [111] |

**Table S5. Terms for other methods in gas-phase action ion spectroscopy**

| Suggested terms | Other terms used in literature |
|---|---|
| **Electronic gas-phase luminescence ion spectroscopy** | Fluorescence spectroscopy [7,34]<br>Luminescence spectroscopy [26,39,42]<br>Gas-phase luminescence spectroscopy [39]<br>Gas-phase luminescence ion spectroscopy [26] |
| **Vibrational laser-induced reaction (LIR) ion spectroscopy** | IR spectroscopy [31]<br>Laser-induced reaction (LIR) spectroscopy [15,31,32] |

**Table S6. Terms for rotational action ion spectroscopy**

| Suggested terms | Other terms used in literature |
|---|---|
| **Laser-induced reaction (LIR) ion spectroscopy** | |
| **Rotational laser induced reaction (LIR) ion spectroscopy** | Pure rotational spectroscopy [33]<br>Rotational spectroscopy [33] |
| **Rotationally resolved vibrational laser induced reaction (LIR) ion spectroscopy** | High-resolution IR spectroscopy [94] |
| **IR-THz laser induced reaction (LIR) ion spectroscopy** | Pure rotational spectroscopy [15]<br>IR-THz double-resonance LIR depletion spectroscopy [15] |
| **Tagging photodissociation ion spectroscopy** | |
| **Rotational laser-induced inhibition of complex growth (LIICG) ion spectroscopy** | Rotational spectroscopy [49] |
| **THz-IR tagging photodissociation ion spectroscopy** | Double resonance rotational spectroscopy [35] |

**Table S7. Notation in names of spectra**

| Name of the technique | Names for the spectra used in literature |
|---|---|
| **Electronic (UV/Vis) photodissociation ion spectroscopy** | Electronic spectrum [3,6,21–23,27,28,43,54,55,57,59,71,73,90,93,96,97,99,100,104,120]<br>UV spectrum [3,5,22,23,25,27,28,38,46,55,59,66,68,69,73,77,79,86,90,95,97,99,104,105,109]<br>Vis spectrum [37]<br>Vibronic spectrum [22]<br>Action spectrum [1,3,23,26,34,39,42]<br>Excitation spectrum [54]<br>Photodissociation spectrum [1,2,4,13,17,37,45,69,79,106,120,121]<br>Photofragment spectrum [44,120]<br>Photofragmentation spectrum [17,27,52,59,60,104]<br>Photodissociation action spectrum [37]<br>Photofragmentation action spectrum [9]<br>Photofragment excitation spectrum [23]<br>Fragmentation action spectrum [9]<br>Laser photodissociation spectrum [2]<br>Electronic action spectrum [100]<br>Electronic absorption spectrum [23,26,120]<br>Electronic excitation spectrum [23,86,105]<br>Electronic fragmentation spectrum [100]<br>Electronic photofragment spectrum [55]<br>Electronic photofragmentation spectrum [57]<br>Electronic photofragment action spectrum [71]<br>Electronic photofragment excitation spectrum [23,55]<br>UV ion spectrum [9]<br>UV action spectrum [3,9,61,67,68]<br>UV excitation spectrum [90,97]<br>UV absorption spectrum [73,77]<br>UV photodissociation (UVPD) spectrum [3,4,25,47,52,66,69,73,110]<br>UV/Vis photodissociation spectrum [2,73]<br>UV photofragment spectrum [5,13,43,44,54,59,79,93,95]<br>UV photofragmentation spectrum [5,13,22,27,28,46,47,59,60,79,90,105,109]<br>VUV/UV photofragmentation spectrum [99]<br>UV fragmentation spectrum [66,86,99]<br>Photofragment UV spectrum [86]<br>Photofragmentation UV spectrum [27,108]<br>UV photofragment excitation spectrum [22,23,105]<br>Vis absorption spectrum [74]<br>Vis photodissociation (VisPD) spectrum [11]<br>Gas-phase action spectrum [3] |

| | |
|---|---|
| | Gas-phase UV spectrum [68]<br>Gas-phase photodissociation spectrum [120]<br>Cold ion spectrum [55]<br>Cold spectrum [55]<br>Cold UV action spectrum [67]<br>Dissapearance spectrum [106]<br>One-color spectrum [110] |
| **2D UV-MS photodissociation ion spectroscopy** | 2D UV-MS spectrum [77,86,108,109]<br>UV-MS spectrum [66,77] |
| **Circular dichroism ion spectroscopy** | Circular dichroism (CD) spectrum [19]<br>Circular dichroism (CD) ion spectrum [19]<br>Electronic circular dichroism (CD) spectrum [19]<br>Gas-phase circular dichroism (CD) spectrum [19] |
| **Electronic UV-UV hole-burning photodissociation ion spectroscopy** | Conformation specific UV spectrum [60,61]<br>UV-UV hole burning spectrum [25,44,60,110]<br>UV-UV pump-probe spectrum [25]<br>UV-UV spectrum [25]<br>Hole-burning spectrum [25,44,60]<br>Pump-probe spectrum [25] |
| **Electronic IR-UV hole-burning photodissociation ion spectroscopy** | IR-UV hole-burning spectrum [21,28]<br>IR-heated UV spectrum [6]<br>Conformationally selective electronic spectrum [44]<br>UV hole-burning spectrum [28] |
| **Electronic IR-UV population transfer photodissociation ion spectroscopy** | IR-UV hole-filling spectrum [79] |
| **Infrared multiple photon dissociation (IRMPD) spectroscopy** | IRMPD spectrum [2,8,14,26,32,41,51,58,59,75,78,83,89,92,103,112]<br>IRMPD action spectrum [83]<br>One-color IRMPD spectrum [112]<br>IRMPD appearance spectrum [36]<br>Resonance-enhanced IRMPD spectrum [37]<br>Vibrational spectrum [32,37,99]<br>IR spectrum [2,8,13,18,23,24,32,38,48,75,91,101]<br>Mid-IR spectrum [51]<br>Fingerprint spectrum [48]<br>Photodissociation spectrum [58,91,101,122] |

| | |
|---|---|
| | Appearance spectrum [36,83]<br>IR fingerprint spectrum [51]<br>Fingerprint IR spectrum [48]<br>Vibrational action spectrum [78]<br>Action IR spectrum [4]<br>IR action spectrum [9]<br>IR photodissociation (IRPD) spectrum [1,32,36,80]<br>IR dissociation spectrum [85]<br>IR photofragmentation spectrum [24]<br>Mid-IR photodissociation spectrum [101]<br>Photodissociaton IR spectrum [101]<br>IR photodepletion spectrum [36]<br>Gas phase IR spectrum [8,51,80,82]<br>IR absorption spectrum [2,91,123]<br>Photofragment excitation spectrum [23]<br>Infrared multiphoton dissociation spectrum [4,13,20,48,80,82,101,104,105]<br>Gas-phase infrared multiphoton dissociation spectrum [20,82]<br>Resonant infrared multiphoton dissociation spectrum [91] |
| **Two-color infrared multiple photon dissociation (IRMPD) spectroscopy** | Two-laser IRMPD spectrum [75]<br>Two-color IRMPD spectrum [112]<br>Isotopomer-selective vibrational spectrum [85]<br>Isotopomer-selective dip spectrum [85]<br>Vibrational spectrum [58]<br>Photodissociation spectrum [58,75]<br>IRLAPS spectrum [58] |
| **Vibrational IR-UV gain photodissociation ion spectroscopy** | Gain spectrum [21,27,28,43,68,79]<br>IR gain spectrum [6,27,28,43,68,79,104]<br>IR photofragment gain spectrum [61]<br>IR-UV gain spectrum [21,79,99,104]<br>IR-VUV gain spectrum [99]<br>IR-UV vibrational gain spectrum [99]<br>IR-UV photodissociation gain spectrum [4]<br>All-conformer IR-UV gain spectrum [21,99]<br>All-conformer gain spectrum [21]<br>Conformer-nonspecific IR gain spectrum [68]<br>Infrared fragment ion gain (IRFIG) spectrum [97]<br>Vibrational spectrum [56]<br>IR spectrum [4,6,97]<br>Conformer non-selective spectrum [79]<br>Non-specific IR spectrum [61] |

| | |
|---|---|
| | Conformation-nonspecific IR spectrum [99]<br>Vibrational absorption spectrum [104]<br>Non-conformation-specific vibrational spectrum [43]<br>IR photodissociation spectrum [110]<br>Linear absorption spectrum [27] |
| **Vibrational IR-UV depletion photodissociation ion spectroscopy** | Depletion spectrum [6,28,59,97,104]<br>Vibrational spectrum [23,28,54,59,79,90,93,104,105]<br>IR spectrum [14,22,23,28,46,54,65,73,77,90,95,96,104,105]<br>Fingerprint spectrum [95]<br>IR depletion spectrum [28,43,93,95]<br>IR dip spectrum [23]<br>IR photodissociation (IRPD) spectrum [110]<br>IR absorption spectrum [73]<br>IR photofragment depletion spectrum [61]<br>IR-UV spectrum [73]<br>IR-UV depletion spectrum [28,43,54,59,67,93,104]<br>IR-UV double resonance spectrum [6,22,23,44,59,73,79,93,105]<br>IR-UV double resonance depletion spectrum [59,90]<br>IR-UV photofragment depletion spectrum [43]<br>Infrared fragment ion dip (IRFID) spectrum [97]<br>Isomer specific spectrum [110]<br>Isomer-specific IR spectrum [95]<br>Conformer-specific spectrum [110]<br>Conformer-specific vibrational spectrum [59,96,99]<br>Conformer-selectrive vibrational spectrum [59,93]<br>Conformer-specific IR spectrum [21,22,54,56,65,67,68,73,90,93,96]<br>Confomer-selective IR spectrum [28,54,56,59]<br>Conformer-selectrive vibrational IR spectrum [59]<br>Conformer-selective IR-UV depletion spectrum [21]<br>Conformer-selective IR-UV double resonance spectrum [59]<br>Conformer-selected IR spectrum [23]<br>Conformation-specific vibrational spectrum [6,43]<br>Conformation-selective vibrational spectrum [59,104]<br>Conformation-specific IR spectrum [6,23,46,55]<br>Conformation-selective IR spectrum [59]<br>Conformation-specific IR depletion spectrum [43]<br>Conformation-selective IR-UV depletion spectrum [59]<br>Conformation-specific infrared fragment ion dip (IRFID) spectrum [97]<br>Single-conformation IR spectrum [61]<br>Linear IR spectrum [93] |

| | |
|---|---|
| | Single-photon absorption action spectrum [59] |
| **Vibrational UV-IR photodissociation ion spectroscopy (IR spectra of ions in electronically excited states)** | Excited-state vibrational spectrum [100]<br>Excited-state IR spectrum [100]<br>Excited-state spectrum [56]<br>IR spectrum [56]<br>Conformer-specific vibrational spectrum [56]<br>Conformer-selective vibrational spectrum [56] |
| **Vibrational IR-UV population transfer photodissociation ion spectroscopy** | IR-induced population transfer spectrum [79]<br>Population transfer spectrum [67] |
| **Vibrational IR-IR-UV hole-burning photodissociation ion spectroscopy** | IR-IR-UV spectrum [21,79]<br>IR-IR-UV gain spectrum [79]<br>IR-IR-VUV gain spectrum [99]<br>IR-IR-UV hole-burning spectrum [99]<br>IR spectrum [79]<br>Conformer-specific spectrum [99]<br>Conformer-specific vibrational spectrum [79]<br>Conformer-resolved vibrational spectrum [79]<br>Conformer-specific IR spectrum [79]<br>Conformer-selective IR spectrum [27]<br>Conformation-specific IR spectrum [99] |
| **Electronic light-induced inhibition of complex growth (LIICG) ion spectroscopy** | Electronic spectrum [16,49,88]<br>Absorption spectrum [16]<br>Electronic absorption spectrum [16]<br>Laser induced spectrum [16] |
| **Electronic (UV/Vis) tagging photodissociation ion spectroscopy** | Electronic spectrum [17,18,50,74]<br>Vis spectrum [17,53]<br>Vibronic spectrum [11]<br>Tagging spectrum [17]<br>Photodissociaton spectrum [17,50]<br>Absoprtion spectrum [12,17,29]<br>Gas-phase spectrum [29]<br>Gas-phase electronic spectrum [10,11]<br>Electronic gas-phase spectrum [10]<br>Tagging UV/Vis spectrum [17]<br>Tagging Vis spectrum [17] |

| | |
|---|---|
| | Vis photodissociation (VisPD) spectrum [17,52,53,74]<br>Vis photofragmentation spectrum [17]<br>Vis predissociation spectrum [52]<br>Tagging vis photodissociation (VisPD) spectrum [17]<br>Tagging visible-light photodissociation spectrum [12]<br>Tagging photodissociation spectrum [17]<br>UV absorption spectrum [12]<br>UV/Vis absorption spectrum [74]<br>UV depletion spectrum [47] |
| **Vibrational (IR) tagging photodissociation ion spectroscopy** | Vibrational spectrum [24,58,64,70,72,85,103,113]<br>IR spectrum [1,10,18,24,30,51,64,74,81,98,102,111,118,119,124]<br>Mid-IR spectrum [117]<br>Tagged spectrum [13]<br>Action spectrum [113]<br>Dissociation spectrum [115]<br>Photodissociation spectrum [58]<br>Predissociation spectrum [41,70,72,78,84,85,116]<br>Photodepletion spectrum [81]<br>Ion depletion spectrum [84]<br>Tagged one-photon spectrum [13]<br>One-photon tagged ion spectrum [13]<br>One-laser spectrum [40]<br>Single-laser spectrum [63]<br>Vibrational fingerprint spectrum [98]<br>Fingerprint IR spectrum [103]<br>Vibrational photodissociation spectrum [117]<br>Vibrational predissociation spectrum [72,84,113,117,118]<br>IR absorption spectrum [18,63,116]<br>IR gas-phase spectrum [10]<br>IR action spectrum [30,41]<br>IR vibrational spectrum [102,103]<br>IR photodissociation (IRPD) spectrum [1,7,17,30,37,51–53,74,78,81,85,119]<br>IR predissociation spectrum [18,40,41,64,76,111]<br>IR photofragmentation spectrum [76]<br>IR vibrational predissociation spectrum [124]<br>Mid-IR action spectrum [119]<br>Tagging IR photodissociation (IRPD) spectrum [7,74,114]<br>Tagging IR predissociation spectrum [111]<br>One-color IR photodissociation (IRPD) spectrum [114]<br>One-photon IR photodissociation (IRPD) spectrum [30]<br>One-laser IR predissociation spectrum [40] |

| | |
|---|---|
| | Single-photon IR photodissociation (IRPD) spectrum [30,51]<br>Single-laser IR predissociation spectrum [40]<br>Cryogenic IR spectrum [98]<br>Messenger predissociation spectrum [70,72]<br>Tagging photodissociation spectrum [53]<br>Tagging photofragmentation spectrum [10]<br>Tag predissociation spectrum [85]<br>Gas phase IR photodissociation (IRPD) spectrum [51]<br>Cold cluster spectrum [85]<br>Non-conformer-specific spectrum [63]<br>Non-isomer-selective spectrum [70,72] |
| **Vibrational laser induced inhibition of complex growth (LIICG) ion spectroscopy** | Laser induced inhibition of complex growth (LIICG) spectrum [18,115]<br>Vibrational spectrum [49]<br>Rovibrational spectrum [49] |
| **Vibrational tagging IR-IR hole-burning photodissociation ion spectroscopy** | Two-color IR-IR isomer selective predissociation spectrum [111]<br>Two-color IR photodissociation (IRPD) spectrum [7]<br>Two-color IR predissociation spectrum [111]<br>Isomer-specific vibrational predissociation spectrum [84]<br>IR-IR ion dip spectrum [40]<br>Structure-specific IR spectrum [40]<br>$IR^2MS^2$ spectrum [84]<br>IR-IR spectrum [40]<br>IR spectrum [79]<br>Fingerprint IR spectrum [63]<br>Vibrational predissociation spectrum [70]<br>Dip spectrum [40,63,70,84]<br>Ion dip spectrum [70,72,84]<br>Conformer-specific spectrum [40,63]<br>Isomer-specific spectrum [84]<br>Isomer-selective spectrum [70,72,84]<br>Double resonance spectrum [63] |
| **Electronic gas-phase luminescence ion spectroscopy** | Emission spectrum [26,42]<br>Reemission spectrum [125]<br>Fluorescence spectrum [17,30,34,39]<br>Fluorescence emission sectrum [34]<br>Fluorescence excitation spectrum [34]<br>Dispersed fluorescence spectrum [39]<br>Luminescence spectrum [39,42] |

| | |
|---|---|
| | Gas-phase luminescence spectrum [42]<br>Light-induce fluorescence spectrum [39]<br>Absorption spectrum [26,52]<br>Excitation spectrum [52] |
| **Vibrational laser induced reaction (LIR) ion spectroscopy** | Laser induced reaction (LIR) spectrum [15,31]<br>Vibrational spectrum [33]<br>IR spectrum [18,31]<br>Action spectrum [33]<br>IR action spectrum [31] |
| **Rotational laser induced reaction (LIR) ion spectroscopy** | Rotational spectrum [33] |
| **Rotationally resolved vibrational laser induced reaction (LIR) ion spectroscopy** | Rotationally resolved excitation spectrum [87]<br>Rovibrational spectrum [33,87] |
| **THz-IR laser induced reaction (LIR) ion spectroscopy** | Pure rotational spectrum [15]<br>Rotational spectrum [15]<br>Two-photon rotational spectrum [15] |
| **THz-IR tagging photodissociation ion spectroscopy** | Predissociation spectrum [35] |
| **Rotational laser induced inhibition of complex growth (LIICG) ion spectroscopy** | Rotational spectrum [49] |

**Bibliography**


1. Duncan MA. Frontiers in the spectroscopy of mass-selected molecular ions. *Int J Mass Spectrom*. 2000;200:545–569.
2. Dunbar RC. Photodissociation of trapped ions. *Int J Mass Spectrom*. 2000;200:571–589.
3. Rosu F, Gabelica V, De Pauw E, Antoine R, Broyer M, Dugourd P. UV spectroscopy of DNA duplex and quadruplex structures in the gas phase. *J Phys Chem A*. 2012;116:5383–5391.
4. Roithova J. Characterization of reaction intermediates by ion spectroscopy. *Chem Soc Rev*. 2012;41:547–559.
5. Redwine JG, Davis ZA, Burke NL, Oglesbee RA, McLuckey SA, Zwier TS. A novel ion trap based tandem mass spectrometer for the spectroscopic study of cold gas phase polyatomic ions. *Int J Mass Spectrom*. 2013;348:9–14.
6. Burke NL, Redwine JG, Dean JC, McLuckey S a., Zwier TS. UV and IR spectroscopy of cold protonated leucine enkephalin. *Int J Mass Spectrom*. 2015;378:196–205.
7. Roithová J, Gray A, Andris E, Jašík J, Gerlich D. Helium Tagging Infrared Photodissociation Spectroscopy of Reactive Ions. *Acc Chem Res*. 2016;49:223–230.
8. Renois-Predelus G, Schindler B, Compagnon I. Analysis of Sulfate Patterns in Glycosaminoglycan Oligosaccharides by MS n Coupled to Infrared Ion Spectroscopy: the Case of GalNAc4S and GalNAc6S. *J Am Soc Mass Spectrom*. 2018;29:1242–1249.
9. Daly S, Porrini M, Rosu F, Gabelica V. Electronic spectroscopy of isolated DNA polyanions. *Faraday Discuss*. 2019;217:361–382.
10. Gerlich D, Jašík J, Roithová J. Tagging fullerene ions with helium in a cryogenic quadrupole trap. *Int J Mass Spectrom*. 2019;438:78–86.
11. Roithová J, Jašík J, Del Pozo Mellado JJ, Gerlich D. Electronic spectra of ions of astrochemical interest: From fast overview spectra to high resolution. *Faraday Discuss*. 2019;217:98–113.
12. Zelenka J, Roithová J. Mechanistic Investigation of Photochemical Reactions by Mass Spectrometry. *ChemBioChem*. 2020;21:1–10.
13. Baer T, Dunbar RC. Ion Spectroscopy: Where Did It Come From; Where Is It Now; and Where Is It Going? *J Am Soc Mass Spectrom*. 2010;21:681–693.
14. Stedwell CN, Galindo JF, Roitberg AE, Polfer NC. Structures of Biomolecular Ions in the Gas Phase Probed by Infrared Light Sources. *Annu Rev Anal Chem*. 2013;6:267–285.
15. Gärtner S, Krieg J, Klemann A, Asvany O, Brünken S, Schlemmer S. High-resolution spectroscopy of CH2D+ in a cold 22-pole ion trap. *J Phys Chem A*. 2013;117:9975–9984.
16. Chakrabarty S, Holz M, Campbell EK, Banerjee A, Gerlich D, Maier JP. A novel method to measure electronic spectra of cold molecular ions. *J Phys Chem Lett*. 2013;4:4051–4054.
17. Navrátil R, Jašík J, Roithová J. Visible photodissociation spectra of gaseous rhodamine ions: Effects of temperature and tagging. *J Mol Spectrosc*. 2017;332:52–58.
18. Gerlich D. Infrared spectroscopy of cold trapped molecular ions using He-tagging. *J Chinese Chem Soc*. 2018;65:637–653.
19. Daly S, Rosu F, Gabelica V. Mass-resolved electronic circular dichroism ion spectroscopy. *Science*.

2020;368:1465–1468.

20. Jašóková L, Hanikýřová E, Schröder D, Roithová J. Aromatic C-H bond activation revealed by infrared multiphoton dissociation spectroscopy. *J Mass Spectrom*. 2012;47:460–465.

21. Pereverzev AY, Szabó I, Kopysov VN, Rosta E, Boyarkin O V. Gas-phase structures reflect the pain-relief potency of enkephalin peptides. *Phys Chem Chem Phys*. 2019;21:22700–22703.

22. Stearns JA, Mercier S, Seaiby C, Guidi M, Boyarkin O V., Rizzo TR. Conformation-Specific Spectroscopy and Photodissociation of Cold, Protonated Tyrosine and Phenylalanine. *J Am Chem Soc*. 2007;129:11814–11820.

23. Rizzo TR, Stearns JA, Boyarkin O V. Spectroscopic Studies of Cold, Gas-Phase Biomolecular Ions. *Int Rev Phys Chem*. 2009;28:481–515.

24. Schöllkopf W, Gewinner S, Junkes H, Paarmann A, von Helden G, Bluem H, Todd AMM. The new IR and THz FEL facility at the Fritz Haber Institute in Berlin. *Adv X-ray Free Lasers Instrum III*. 2015;9512:95121L.

25. Inokuchi Y, Nakatsuma M, Kida M, Ebata T. Conformation of Alkali Metal Ion-Benzo-12-Crown-4 Complexes Investigated by UV Photodissociation and UV-UV Hole-Burning Spectroscopy. *J Phys Chem A*. 2016;120:6394–6401.

26. Stockett MH, Houmøller J, Brøndsted Nielsen S. Nile blue shows its true colors in gas-phase absorption and luminescence ion spectroscopy. *J Chem Phys*. 2016;145:104303.

27. Pereverzev AY, Kopysov V, Boyarkin OV. High Susceptibility of Histidine to Charge Solvation Revealed by Cold Ion Spectroscopy. *Angew Chemie - Int Ed*. 2017;56:15639–15643.

28. Pereverzev AY, Boyarkin O V. Exploring the relevance of gas-phase structures to biology : cold ion spectroscopy of the decapeptide neurokinin A. *Phys Chem Chem Phys*. 2017;19:3468–3472.

29. Gatchell M, Martini P, Laimer F, Goulart M, Calvo F, Scheier P. Spectroscopy of corannulene cations in helium nanodroplets. *Faraday Discuss*. 2019;217:276–289.

30. Danell AS, Parks JH. FRET measurements of trapped oligonucleotide duplexes. *Int J Mass Spectrom*. 2003;229:35–45.

31. Schlemmer S, Asvany O, Giesen T. Comparison of the cis-bending and C-H stretching vibration on the reaction of C2H2+ with H2 using laser induced reactions. *Phys Chem Chem Phys*. 2005;7:1592–1600.

32. Oomens J, Sartakov BG, Meijer G, von Helden G. Gas-phase infrared multiple photon dissociation spectroscopy of mass-selected molecular ions. *Int J Mass Spectrom*. 2006;254:1–19.

33. Asvany O, Ricken O, Müller HSP, Wiedner MC, Giesen TF, Schlemmer S. High-resolution rotational spectroscopy in a cold ion trap: H2D+ and D2H+. *Phys Rev Lett*. 2008;100:13–16.

34. Daly S, Poussigue F, Simon AL, Macaleese L, Bertorelle F, Chirot F, Antoine R, Dugourd P. Action-FRET: Probing the molecular conformation of mass-selected gas-phase peptides with förster resonance energy transfer detected by acceptor-specific fragmentation. *Anal Chem*. 2014;86:8798–8804.

35. Töpfer M, Salomon T, Kohguchi H, Dopfer O, Yamada KMT, Schlemmer S, Asvany O. Double Resonance Rotational Spectroscopy of Weakly Bound Ionic Complexes: The Case of Floppy CH3+ -He. *Phys Rev Lett*. 2018;121:143001.

36. Polfer NC, Oomens J, Suhai S, Paizs B. Infrared spectroscopy and theoretical studies on gas-phase protonated Leu-enkephalin and its fragments: Direct experimental evidence for the mobile proton. *J Am Chem Soc*. 2007;129:5887–5897.

37. Duncan MA. Structures, energetics and spectroscopy of gas phase transition metal ion-benzene complexes. *Int J Mass Spectrom*. 2008;272:99–118.

38. Flórez AIG, Ahn D-S, Gewinner S, Schöllkopf W, von Helden G. IR spectroscopy of protonated leu-enkephalin and its 18-crown-6 complex embedded in helium droplets. *Phys Chem Chem Phys*. 2015;17:21902–21911.

39. Stockett MH, Houmøller J, Støchkel K, Svendsen A, Brøndsted Nielsen S. A cylindrical quadrupole ion trap in combination with an electrospray ion source for gas-phase luminescence and absorption spectroscopy. *Rev Sci Instrum*. 2016;87:053103.

40. Voss JM, Kregel SJ, Fischer KC, Garand E. IR-IR Conformation Specific Spectroscopy of Na + (Glucose) Adducts. *J Am Soc Mass Spectrom*. 2018;29:42–50.

41. Jusko P, Brünken S, Asvany O, Thorwirth S, Stoffels A, Van Der Meer L, Berden G, Redlich B, Oomens J, Schlemmer S. The FELion cryogenic ion trap beam line at the FELIX free-electron laser laboratory: Infrared signatures of primary alcohol cations. *Faraday Discuss*. 2019;217:172–202.

42. Kjær C, Nielsen SB. Luminescence spectroscopy of oxazine dye cations isolated in vacuo. *Phys Chem Chem Phys*. 2019;21:4600–4605.

43. Burke NL, DeBlase AF, Redwine JG, Hopkins JR, McLuckey SA, Zwier TS. Gas-Phase Folding of a Prototypical Protonated Pentapeptide: Spectroscopic Evidence for Formation of a Charge-Stabilized β-Hairpin. *J Am Chem Soc*. 2016;138:2849–2857.

44. Choi CM, Choi DH, Heo J, Kim NJ, Kim SK. Ultraviolet-ultraviolet hole burning spectroscopy in a quadrupole ion trap: Dibenzo[18]crown-6 complexes with alkali metal cations. *Angew Chemie - Int Ed*. 2012;51:7297–7300.

45. Talbot FO, Tabarin T, Antoine R, Broyer M, Dugourd P. Photodissociation spectroscopy of trapped protonated tryptophan. *J Chem Phys*. 2005;122:074310.

46. Stearns JA, Boyarkin O V., Rizzo TR. Effects of N-terminus substitution on the structure and spectroscopy of gas-phase helices. *Chimia (Aarau)*. 2008;62:240–243.

47. Boyarkin O V., Kopysov V. Cryogenically cooled octupole ion trap for spectroscopy of biomolecular ions. *Rev Sci Instrum*. 2014;85:033105.

48. Jašíková L, Roithová J. Infrared Multiphoton Dissociation Spectroscopy with Free-Electron Lasers: On the Road from Small Molecules to Biomolecules. *Chem - A Eur J*. 2018;24:3374–3390.

49. Brünken S, Kluge L, Stoffels A, Asvany O, Schlemmer S. Laboratory rotational spectrum of l-C3H+ and confirmation of its astronomical detection. *Astrophys J Lett*. 2014;783:1–5.

50. Bieske EJ, Soliva A, Welker MA, Maier JP. The B<-X electronic spectrum of N 2+ –He. *J Chem Phys*. 1990;93:4477–4478.

51. MacAleese L, Maître P. Infrared spectroscopy of organometallic ions in the gas phase: From model to real world complexes. *Mass Spectrom Rev*. 2007;26:583–605.

52. Jašík J, Navrátil R, Němec I, Roithová J. Infrared and Visible Photodissociation Spectra of Rhodamine Ions at 3 K in the Gas Phase. *J Phys Chem A*. 2015;119:12648–12655.

53. Zelenka J, Cibulka R, Roithová J. Flavinium Catalysed Photooxidation: Detection and Characterization of Elusive Peroxyflavinium Intermediates. *Angew Chemie - Int Ed*. 2019;58:15412–15420.

54. Guidi M, Lorenz UJ, Papadopoulos G, Boyarkin O V., Rizzo TR. Spectroscopy of Protonated Peptides Assisted by Infrared Multiple Photon Excitation. *J Phys Chem A*. 2009;113:797–799.

55. Papadopoulos G, Svendsen A, Boyarkin O V, Rizzo TR. Spectroscopy of mobility-selected biomolecular ions. *Faraday Discuss*. 2011;150:243–255.

56. Zabuga A V., Kamrath MZ, Boyarkin O V., Rizzo TR. Fragmentation mechanism of UV-excited peptides in the gas phase. *J Chem Phys*. 2014;141:154309.

57. Boyarkin O V., Mercier SR, Kamariotis A, Rizzo TR. Electronic Spectroscopy of Cold, Protonated Tryptophan and Tyrosine. *J Am Chem Soc*. 2006;128:2816–2817.

58. Altinay G, Metz RB. Comparison of IRMPD, Ar-tagging and IRLAPS for vibrational spectroscopy of Ag+(CH3OH). *Int J Mass Spectrom*. 2010;297:41–45.

59. Wassermann TN, Boyarkin O V, Paizs B, Rizzo TR. Conformation-Specific Spectroscopy of Peptide Fragment Ions in a Low-Temperature Ion Trap. *J Am Chem Soc Mass Spectrom*. 2012;23:1029–1045.

60. Kang H, Féraud G, Dedonder-Lardeux C, Jouvet C. New method for double-resonance spectroscopy in a cold quadrupole ion trap and its application to UV-UV hole-burning spectroscopy of protonated adenine dimer. *J Phys Chem Lett*. 2014;5:2760–2764.

61. DeBlase AF, Harrilal CP, Lawler JT, Burke NL, McLuckey SA, Zwier TS. Conformation-Specific Infrared and Ultraviolet Spectroscopy of Cold [YAPAA+H]+ and [YGPAA+H]+ Ions: A Stereochemical “Twist” on the β-Hairpin Turn. *J Am Chem Soc*. 2017;139:5481–5493.

62. Spieler S, Kuhn M, Postler J, Simpson M, Wester R, Scheier P, Ubachs W, Bacalla X, Bouwman J, Linnartz H. C60 + and the Diffuse Interstellar Bands: An Independent Laboratory Check. *Astrophys J*. 2017;846:168.

63. Scutelnic V, Rizzo TR. Cryogenic Ion Spectroscopy for Identification of Monosaccharide Anomers. *J Phys Chem A*. 2019;123:2815–2819.

64. Masson A, Kamrath MZ, Perez MAS, Glover MS, Rothlisberger U, Clemmer DE, Rizzo TR. Infrared Spectroscopy of Mobility-Selected H+-Gly-Pro-Gly-Gly (GPGG). *J Am Soc Mass Spectrom*. 2015;26:1444–1454.

65. Roy TK, Kopysov V, Nagornova NS, Rizzo TR, Boyarkin O V., Gerber RB. Conformational Structures of a Decapeptide Validated by First Principles Calculations and Cold Ion Spectroscopy. *ChemPhysChem*. 2015;16:1374–1378.

66. Kopysov V, Boyarkin O V. Resonance Energy Transfer Relates the Gas-Phase Structure and Pharmacological Activity of Opioid Peptides. *Angew Chemie - Int Ed*. 2016;55:689–692.

67. Harrilal CP, DeBlase AF, Fischer JL, Lawler JT, McLuckey SA, Zwier TS. Infrared Population Transfer Spectroscopy of Cryo-Cooled Ions: Quantitative Tests of the Effects of Collisional Cooling on the Room Temperature Conformer Populations. *J Phys Chem A*. 2018;122:2096–2107.

68. Ujma J, Kopysov V, Nagornova NS, Migas LG, Lizio MG, Blanch EW, MacPhee C, Boyarkin O V., Barran PE. Initial Steps of Amyloidogenic Peptide Assembly Revealed by Cold-Ion Spectroscopy. *Angew Chemie - Int Ed*. 2018;57:213–217.

69. Pereverzev AY, Koczor-Benda Z, Saparbaev E, Kopysov VN, Rosta E, Boyarkin O V. Spectroscopic Evidence for Peptide-Bond-Selective Ultraviolet Photodissociation. *J Phys Chem Lett*. 2020;11:206–209.

70. Elliott BM, Relph RA, Roscioli JR, Bopp JC, Gardenier GH, Guasco TL, Johnson MA. Isolating the spectra of cluster ion isomers using Ar-"tag" -mediated IR-IR double resonance within the vibrational manifolds: Application to NO2- H2O. *J Chem Phys*. 2008;129:094303.

71. Mercier SR, Boyarkin O V., Kamariotis A, Guglielmi M, Tavernelli I, Cascella M, Rothlisberger U, Rizzo TR. Microsolvation Effects on the Excited-State Dynamics of Protonated Tryptophan. *J Am Chem Soc*. 2006;128:16938–16943.

72. Elliott BM, Relph RA, Roscioli JR, Bopp JC, Gardenier GH, Guasco TL, Johnson MA. Isolating the spectra of cluster ion isomers using Ar-"tag" -mediated IR-IR double resonance within the vibrational manifolds: Application to NO2- *H2O. *J Chem Phys*. 2008;129:094303.

73. Inokuchi Y, Boyarkin O V., Kusaka R, Haino T, Ebata T, Rizzo TR. UV and IR Spectroscopic Studies of Cold Alkali Metal Ion-Crown Ether Complexes in the Gas Phase. *J Am Chem Soc*. 2011;133:12256–12263.

74. Navrátil R, Wiedbrauk S, Jašík J, Dube H, Roithová J. Transforming hemithioindigo from a two-way to a one-way molecular photoswitch by isolation in the gas phase. *Phys Chem Chem Phys*. 2018;20:6868–6876.

75. Watson CH, Zimmerman JA, Bruce JE, Eyler JR. Resonance-enhanced two-laser infrared multiple photon dissociation of gaseous ions. *J Phys Chem*. 1991;95:6081–6086.

76. Gerlich D, Jašík J, Andris E, Navrátil R, Roithová J. Collisions of FeO+ with H2 and He in a Cryogenic Ion Trap. *ChemPhysChem*. 2016;17:3723–3739.

77. Kopysov V, Makarov A, Boyarkin O V. Nonstatistical UV Fragmentation of Gas-Phase Peptides Reveals Conformers and Their Structural Features. *J Phys Chem Lett*. 2016;7:1067–1071.

78. Heine N, Asmis KR. Cryogenic ion trap vibrational spectroscopy of hydrogen-bonded clusters relevant to atmospheric chemistry. *Int Rev Phys Chem*. 2015;34:1–34.

79. Pereverzev AY, Cheng X, Nagornova NS, Reese DL, Steele RP, Boyarkin O V. Vibrational Signatures of Conformer-Specific Intramolecular Interactions in Protonated Tryptophan. *J Phys Chem A*. 2016;120:5598−5608.

80. Asmis KR, Pivonka NL, Santambrogio G, Brümmer M, Kaposta C, Neumark DM, Wöste L. Gas-Phase Infrared Spectrum of the Protonated Water Dimer. *Science (80- )*. 2003;299:1375–1377.

81. Brümmer M, Kaposta C, Santambrogio G, Asmis KR. Formation and photodepletion of cluster ion-messenger atom complexes in a cold ion trap: Infrared spectroscopy of VO+, VO2+, and VO3+. *J Chem Phys*. 2003;119:12700–12703.

82. Jagoda-Cwiklik B, Jungwirth P, Rulíšek L, Milko P, Roithová J, Lemaire J, Maitre P, Ortega JM, Schröder D. Micro-Pydration of the MgNO3+ Cation in the Gas Phase. *ChemPhysChem*. 2007;8:1629–1639.

83. Citir M, Hinton CS, Oomens J, Steill JD, Armentrout PB. Infrared multiple photon dissociation spectroscopy of protonated histidine and 4-phenyl imidazole. *Int J Mass Spectrom*. 2012;330–332:6–15.

84. Leavitt CM, Wolk AB, Fournier JA, Kamrath MZ, Garand E, Van Stipdonk MJ, Johnson MA. Isomer-

Specific IR–IR Double Resonance Spectroscopy of D 2 -Tagged Protonated Dipeptides Prepared in a Cryogenic Ion Trap. *J Phys Chem Lett*. 2012;3:1099–1105.

85. Yang N, Duong CH, Kelleher PJ, Johnson MA, McCoy AB. Isolation of site-specific anharmonicities of individual water molecules in the I−·(H2O)2 complex using tag-free, isotopomer selective IR-IR double resonance. *Chem Phys Lett*. 2017;690:159–171.

86. Saparbaev E, Kopysov V, Yamaletdinov R, Pereverzev AY, Boyarkin O V. Interplay of H-Bonds with Aromatics in Isolated Complexes Identifies Isomeric Carbohydrates. *Angew Chemie - Int Ed*. 2019;58:7346–7350.

87. Schlemmer S, Kuhn T, Lescop E, Gerlich D. Laser excited N2+ in a 22-pole ion trap:: Experimental studies of rotational relaxation processes. *Int J Mass Spectrom*. 1999;185:589–602.

88. Asvany O, Brünken S, Kluge L, Schlemmer S. COLTRAP: A 22-pole ion trapping machine for spectroscopy at 4 K. *Appl Phys B Lasers Opt*. 2014;114:203–211.

89. Maitre P, Scuderi D, Corinti D, Chiavarino B, Crestoni ME, Fornarini S. Applications of Infrared Multiple Photon Dissociation (IRMPD) to the Detection of Posttranslational Modifications. *Chem Rev*. 2020;120:3261–3295.

90. Stearns JA, Boyarkin O V., Rizzo TR. Spectroscopic Signatures of Gas-Phase Helices: Ac-Phe-(Ala)5-Lys-H+ and Ac-Phe-(Ala)10-Lys-H+. *J Am Chem Soc Commun*. 2007;129:13820–13821.

91. Grégoire G, Gaigeot MP, Marinica DC, Lemaire J, Schermann JP, Desfrançois C. Resonant infrared multiphoton dissociation spectroscopy of gas-phase protonated peptides. Experiments and Car-Parrinello dynamics at 300 K. *Phys Chem Chem Phys*. 2007;9:3082–3097.

92. Correia CF, Balaj PO, Scuderi D, Maitre P, Ohanessian G. Vibrational signatures of protonated, phosphorylated amino acids in the gas phase. *J Am Chem Soc*. 2008;130:3359–3370.

93. Nagornova NS, Rizzo TR, Boyarkin O V. Highly Resolved Spectra of Gas-Phase Gramicidin S : A Benchmark for Peptide Structure Calculations. *J Am Chem Soc*. 2010;132:4040–4041.

94. Gärtner S, Krieg J, Klemann A, Asvany O, Schlemmer S. Rotational transitions of CH2D+ determined by high-resolution IR spectroscopy. *Astron Astrophys*. 2010;516:L3.

95. Lorenz UJ, Rizzo TR. Multiple Isomers and Protonation Sites of the Phenylalanine/Serine Dimer. *J Am Chem Soc*. 2012;134:11053–11055.

96. Carr JK, Zabuga A V., Roy S, Rizzo TR, Skinner JL. Assessment of Amide I Spectroscopic Maps for a Gas-Phase Peptide Using IR-UV Double-Resonance Spectroscopy and Density Functional Theory Calculations. *J Chem Phys*. 2014;140:224111.

97. Dean JC, Burke NL, Hopkins JR, Redwine JG, Ramachandran P V., McLuckey SA, Zwier TS. UV Photofragmentation and IR Spectroscopy of Cold, G-Type β-O-4 and β-β Dilignol-Alkali Metal Complexes: Structure and Linkage-Dependent Photofragmentation. *J Phys Chem A*. 2015;119:1917–1932.

98. Khanal N, Masellis C, Kamrath MZ, Clemmer DE, Rizzo TR. Cryogenic IR spectroscopy combined with ion mobility spectrometry for the analysis of human milk oligosaccharides. *Analyst*. 2018;143:1846–1852.

99. Pereverzev AY, Kopysov VN, Boyarkin O V. Peptide Bond Ultraviolet Absorption Enables Vibrational Cold-Ion Spectroscopy of Nonaromatic Peptides. *J Phys Chem Lett*. 2018;9:5262–5266.

100. Scutelnic V, Prlj A, Zabuga A, Corminboeuf C, Rizzo TR. Infrared Spectroscopy as a Probe of Electronic Energy Transfer. *J Phys Chem Lett*. 2018;9:3217–3223.

101. Pagel K, Kupser P, Bierau F, Polfer NC, Steill JD, Oomens J, Meijer G, Koksch B, von Helden G. Gas-phase IR spectra of intact alfa-helical coiled coil protein complexes. *Int J Mass Spectrom*. 2009;283:161–168.

102. Warnke S, Ben Faleh A, Pellegrinelli RP, Yalovenko N, Rizzo TR. Combining ultra-high resolution ion mobility spectrometry with cryogenic IR spectroscopy for the study of biomolecular ions. *Faraday Discuss*. 2019;217:114–125.

103. Ben Faleh A, Warnke S, Rizzo TR. Combining Ultrahigh-Resolution Ion-Mobility Spectrometry with Cryogenic Infrared Spectroscopy for the Analysis of Glycan Mixtures. *Anal Chem*. 2019;91:4876–4882.

104. Nagornova NS, Rizzo TR, Boyarkin O V. Exploring the Mechanism of IR-UV Double-Resonance for Quantitative Spectroscopy of Protonated Polypeptides and Proteins. *Angew Chemie - Int Ed*. 2013;52:6002–6005.

105. Stearns JA, Seaiby C, Boyarkin O V., Rizzo TR. Spectroscopy and conformational preferences of gas-phase helices. *Phys Chem Chem Phys*. 2009;11:125–132.

106. Dunbar RC, Fu EW. Photodissociation Spectroscopy of Gaseous C7H8+ Cations. *J Am Chem Soc*. 1973;95:2716–2718.

107. Dunbar RC, Hays JD, Honovich JP, Lev NB. Two-Laser Multiphoton Dissociation of Iodobenzene Cation. *J Am Chem Soc*. 1980;102:3950–3951.

108. Solovyeva EM, Kopysov VN, Pereverzev AY, Lobas AA, Moshkovskii SA, Gorshkov M V., Boyarkin O V. Method for Identification of Threonine Isoforms in Peptides by Ultraviolet Photofragmentation of Cold Ions. *Anal Chem*. 2019;doi:10.1021/acs.analchem.9b00770.

109. Kopysov V, Gorshkov M V., Boyarkin O V. Identification of isoforms of aspartic acid residues in peptides by 2D UV-MS fingerprinting of cold ions. *Analyst*. 2018;143:833–836.

110. Féraud G, Dedonder C, Jouvet C, Inokuchi Y, Haino T, Sekiya R, Ebata T. Development of ultraviolet-ultraviolet hole-burning spectroscopy for cold gas-phase ions. *J Phys Chem Lett*. 2014;5:1236–1240.

111. Jašík J, Gerlich D, Roithová J. Two-color infrared predissociation spectroscopy of C6H62+ isomers using helium tagging. *J Phys Chem A*. 2015;119:2532–2542.

112. Nosenko Y, Menges F, Riehn C, Niedner-Schatteburg G. Investigation by two-color IR dissociation spectroscopy of Hoogsteen-type binding in a metalated nucleobase pair mimic. *Phys Chem Chem Phys*. 2013;15:8171–8178.

113. Gerardi HK, Gardenier GH, Viswanathan U, Auerbach SM, Johnson MA. Vibrational predissociation spectroscopy and theory of Ar-tagged, protonated Imidazole (Im) Im1-3H+.Ar clusters. *Chem Phys Lett*. 2011;501:172–178.

114. Andris E, Jašík J, Gómez L, Costas M, Roithová J. Spectroscopic Characterization and Reactivity of Triplet and Quintet Iron(IV) Oxo Complexes in the Gas Phase. *Angew Chemie*. 2016;128:3701–3705.

115. Savić I, Gerlich D, Asvany O, Jusko P, Schlemmer S. Controlled synthesis and analysis of He-H+3 in a 3.7 K ion trap. *Mol Phys*. 2015;113:2320–2332.

116. Okumura M, Yeh LI, Lee YT. The vibrational predissociation spectroscopy of hydrogen cluster ions. *J Chem Phys*. 1985;83:3705–3706.

117. Nizkorodov SA, Dopfer O, Meuwly M, Maier JP, Bieske EJ. Mid-infrared spectra of the proton-bound complexes Nen–HCO+ (n=1,2). *J Chem Phys*. 1996;105:1770–1777.

118. Niemann T, Strate A, Ludwig R, Zeng HJ, Menges FS, Johnson MA. Cooperatively enhanced hydrogen bonds in ionic liquids: Closing the loop with molecular mimics of hydroxy-functionalized cations. *Phys Chem Chem Phys*. 2019;21:18092–18098.

119. Franke PR, Duncan MA, Douberly GE. Infrared photodissociation spectroscopy and anharmonic vibrational study of the HO4+ molecular ion. *J Chem Phys*. 2020;152:174309.

120. Dzhonson A, Gerlich D, Bieske EJ, Maier JP. Apparatus for the study of electronic spectra of collisionally cooled cations: para-dichlorobenzene. *J Mol Struct*. 2006;795:93–97.

121. Dunbar RC. Photodissociation of Toluene Parent Cations. *J Am Chem Soc*. 1973;95:472–476.

122. Bomse DS, Woodin RL, Beauchamp JL. Molecular Activation with Low-Intensity CW Infrared Laser Radiation. Multiphoton Dissociation of Ions Derived from Diethyl Ether. *J Am Chem Soc*. 1979;101:5503–5512.

123. Woodin RL, Bomse DS, Beauchamp JL. Multiphoton Dissociation of Molecules with Low Power Continuous Wave Infrared Laser Radiation. *J Am Chem Soc*. 1978;100:3248–3250.

124. Okumura M, Yeh LI, Myers JD, Lee YT. Infrared spectra of the cluster ions H7O3+·H2 and H9O4+·H2. *J Chem Phys*. 1986;85:2328–2329.

125. Neuhauser W, Hohenstatt M, Toschek P, Dehmelt H. Optical-Sideband Cooling of Visible Atom Cloud Confined in Parabolic Well. *Phys Rev Lett*. 1978;41:233–236.
